# The WW domains dictate isoform-specific regulation of YAP1 stability and pancreatic cancer cell malignancy

**DOI:** 10.7150/thno.42795

**Published:** 2020-03-15

**Authors:** Qiang Guo, Meiyu Quan, Jinglai Dong, Jing Bai, Jie Wang, Rui Han, Wei Wang, Yaxin Cai, Yu-Qing Lv, Qianjie Chen, Huijing Xu, Han-Deng Lyu, Liancheng Deng, Depu Zhou, Xueyuan Xiao, Stijn De Langhe, Daniel D. Billadeau, Zhenkun Lou, Jin-San Zhang

**Affiliations:** 1 School of Pharmaceutical Sciences, Wenzhou Medical University, Wenzhou, Zhejiang 325035, China; 2 Center for Precision Medicine, the First Affiliated Hospital of Wenzhou Medical University, Wenzhou, Zhejiang 325000, China; 3 Key Laboratory of Cell Proliferation and Regulation Biology, Ministry of Education, Beijing Normal University, Beijing 100875, China; 4 Department of Medicine, Division of Pulmonary, Allergy and Critical Care Medicine, University of Alabama at Birmingham, Birmingham, 35294-2182 AL, USA; 5 Division of Oncology Research, and Schulze Center for Novel Therapeutics, Mayo Clinic, Rochester, MN 55905, USA; 6 Institute of Life Sciences, Wenzhou University, Wenzhou, Zhejiang 325035, China

**Keywords:** YAP1, isoform, pancreatic cancer, WW domain, LATS1, protein stability

## Abstract

YAP1 is a key mediator of the Hippo pathway capable of exerting a profound effect on organ size as well as tumorigenesis. Alternative mRNA splicing of human YAP1 results in at least 8 protein isoforms that differ within the 2^nd^ WW motif and the transcriptional activation domain.

**Methods**: To investigate the isoform-specific differences in their mRNA expression, transcriptional activity and tumor-promoting function, we cloned cDNA encoding all of the eight YAP1 protein isoforms. Then, we examined their mRNA expression, subcellular localization, transcriptional regulation properties, interactions with key regulatory partners, and protein stability in response to changes in cell density, as well as their effects on pancreatic cancer cell malignancy both *in vitro* and *in vivo*.

**Results**: Multiple YAP1 mRNA isoforms are expressed in commonly used pancreatic cancer lines as well as human pancreatic cancer PDX lines. Based on the analysis of heterologous reporter and endogenous target genes, all YAP1 isoforms are capable of activating transcription, albeit to a different extent. Importantly, we unveiled a marked discrepancy between the mRNA and protein expression levels of the YAP1-1 and YAP1-2 isoforms. We further discovered that the YAP1-2 isoform, which contains two tandem WW motifs, is less stable at the protein level, particularly at high cell densities. Mechanistically, we found that the presence of the 2^nd^ WW motif in YAP1-2 facilitates the *de novo* formation of the YAP1-2/AMOT/LATS1 complex and contributes to a stronger binding of YAP1-2 to LATS1 and subsequently increased YAP1-2 ubiquitination and degradation by β-TRCP.

**Conclusion**: Our data reveals a potent effect of YAP1-1 on pancreatic cancer malignancy *in vitro* and *in vivo* and provides novel mechanistic insight into isoform-specific and cell density-dependent regulation of YAP1 stability, as well as its impact on cancer malignancy.

## Introduction

Yes-associated protein 1 (YAP1) is a major downstream effector of the Hippo pathway, which plays crucial roles in organ size control and tissue homeostasis. Hippo signaling is also an established tumor suppressor pathway, whereas YAP1 has been identified as an oncogene in various malignant tumors. The overexpression of YAP1 during embryonic development leads to oversized organs and eventual tumor formation 1. In mammals, the deregulation of Hippo/YAP1 has been linked to several human cancers, including colorectal cancer 2, lung cancer 3, liver cancer 4, breast cancer 5, renal cell carcinoma 6 and pancreatic adenocarcinoma (PDAC) 7. YAP1 overexpression is detected during early stages of pancreatic carcinogenesis 8 and represents one of the key factors in embryonic development that is reactivated during tumorigenesis 9, 10. Interestingly, YAP1 has been implicated in both oncogenic KRAS-dependent and -independent cancer-promoting activities 11-14, suggesting it plays a critical role across a wide range of cancers. The activity of YAP1 regulates several key cellular properties linked to tumorigenesis, including cell proliferation, survival, stem cell maintenance, epithelial-mesenchymal transition (EMT), and metastasis 9.

**Figure 1:**
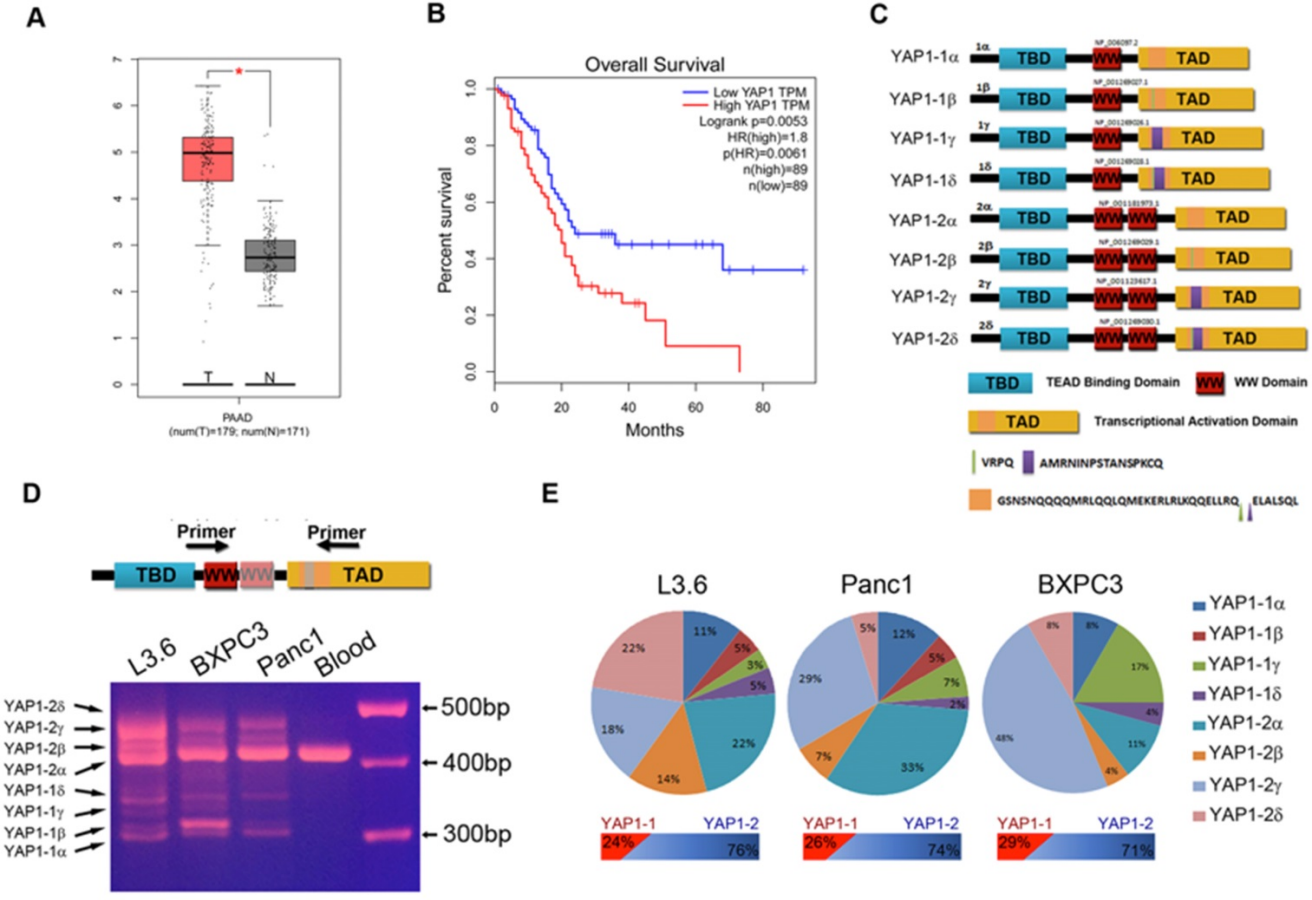
** Characterization of YAP1 expression in PDAC tissue samples and cell lines. (A)** The transcriptional profile of YAP1 was analyzed in 179 pancreatic cancer tissue samples (T) and 171 normal tissue samples (N) obtained from PAAD datasets in TCGA. **(B)** Patients with high YAP1 expression (n=89) had poorer overall survival (OS) rate than those with low YAP1 expression (n = 89). Long-rank p=0.0056. **(C)** Schematic representation of the eight isoforms of YAP1. **(D)** PCR products amplified from the cDNA of human pancreatic cancer cell lines, with peripheral blood mononuclear cells used as a control. **(E)** Calculated percentage of each isoform in the different pancreatic cancer cell lines based on direct sequencing of T-vector clones.

The human *YAP1* gene, upon alternative mRNA splicing, generates at least eight protein isoforms that differ in the regions of the 2^nd^ WW domain and transcriptional activation domain (TAD) 15. The WW domain(s) are responsible for protein-protein interactions, while the TAD governs the transcriptional activity of YAP1. Based on the number of WW domains present, YAP1 can be separated into two subgroups: YAP1-1 (with one WW domain) and YAP1-2 (with two WW domains). Each of YAP1 subgroups can be further divided into four subtypes, namely α, β, γ and δ based on the alternative splicing within the TAD (Figure 1C). A recent study on YAP isoforms with a focus on the TAD and transcriptional potency showed that isoform-specific insertions within the YAP1 leucine zipper have a negative effect on transcriptional activity 16.

The WW domain consists of an imperfect repeat of 30-40 amino acid residues with two invariant tryptophan residues that mediate specific interactions with partners containing short proline-rich sequences 17, 18. The WW domain of YAP1 is involved in complex formation with a number of PPxY motif-containing proteins in the Hippo pathway 19, such as LATS1/2 1, AMOT 20, WBP2, and PTPN14. The presence of single or double WW domains may influence the interaction of YAP1 with these proteins. It has been demonstrated that YAP1-1, which contains one WW domain, cannot interact with AMOT 21. The downregulation of YAP1 by LATS1/2 also depends on its interaction with the WW domain 22. It has been suggested that the two WW domains of YAP1 behave as independent units with different binding preferences 23, but the 2^nd^ WW domain seems to have much less impact on transcriptional activity than the TAD insertions 16. The role of the 2^nd^ WW domain in regulating YAP1 biological and functional properties remains incompletely understood.

In this study, we determined the relative expression of YAP1 mRNA isoforms in human PDAC cells, and cloned cDNAs encoding the full-length protein of all 8 YAP1 isoforms. Taking advantage of this full panel of YAP1 expression vectors, we derived a comprehensive panel of knockout and reconstituted stable cell lines and systematically investigated the differences in the regulation and functional properties of each YAP1 isoform. Our results revealed a major discrepancy between the mRNA and protein expression of the YAP1-1 and YAP1-2 subtypes and the critical role of the 2^nd^ WW domain in dictating the isoform-specific cell density-dependent regulation of YAP1 stability and its impact on cell proliferation.

## Results

### PDAC cells mainly express YAP1-2 mRNA isoforms

YAP1 expression was much higher in the PDAC patient sample (T) than in the normal sample (N) (Figure 1A). Kaplan-Meier analysis and log-rank test show that the survival of patients with high YAP1 expression was significantly lower than in those with low YAP1 expression (Figure 1B). Alternative splicing of the human YAP1 gene generates at least eight mRNA isoforms (Figure 1C and Supplement Figure 1) 15, 24. We performed RT-PCR with YAP1 specific primers flanking the alternatively spliced regions and cDNA from indicated PDAC cell lines and pancreatic cancer PDX cells, with peripheral blood mononuclear cells (PBMCs) as a control (Figure 1D and Supplement Figure 3A). Results showed that PDAC cells express multiple YAP1 isoforms at variable levels, whereas normal PBMCs only express a single YAP1 isoform, which was further confirmed as YAP1-2α by direct sequencing (data not shown). However, isoforms β and δ differ from α and γ by only 12 bp (Figure 1D, bottom half), the PCR products could not be separated into distinct bands, we therefore cloned the PCR products into a T-vector and selected 100 clones with inserts for direct sequencing analysis and calculated the percentage of each isoform relative to the total (Figure 1E). All 8 isoforms, except YAP1-1β, which appeared to be absent in BxPC3, were expressed in PDAC cell lines at various levels. Overall, YAP1-2 mRNA isoforms were more dominant than those of YAP1-1 isoforms, while the proportions of isoforms α, β, γ and δ varied in different cell lines (Figure 1E).

We employed an overlapping PCR approach to generate full-length ORF-encoding cDNAs for all 8 isoforms and have them cloned into a eukaryotic vector with an N-terminal Flag tag. Immunoblot analysis of transfected HEK293 cell lysate showed that all 8 isoforms were successfully constructed and expressed proteins of the predicted sizes (Supplement Figure 2A). To facilitate isoform-specific characterization, we further subcloned these YAP1 cDNA into a lentiviral expression vector and generated, in YAP1-KO background (Supplement Figure 2B), reconstituted expression of YAP1 isoforms by lentiviral infection (Supplement Figure 2C) with their relative expression confirmed by qRT-PCR (Supplement Figure 2D). Each reconstituted L3.6-YAP1 stable cell line was confirmed to express one single type of YAP1 by RT-PCR (Supplement Figure 2E).

### TA domain insertions negatively impact YAP1 transcriptional activity

**Figure 2:**
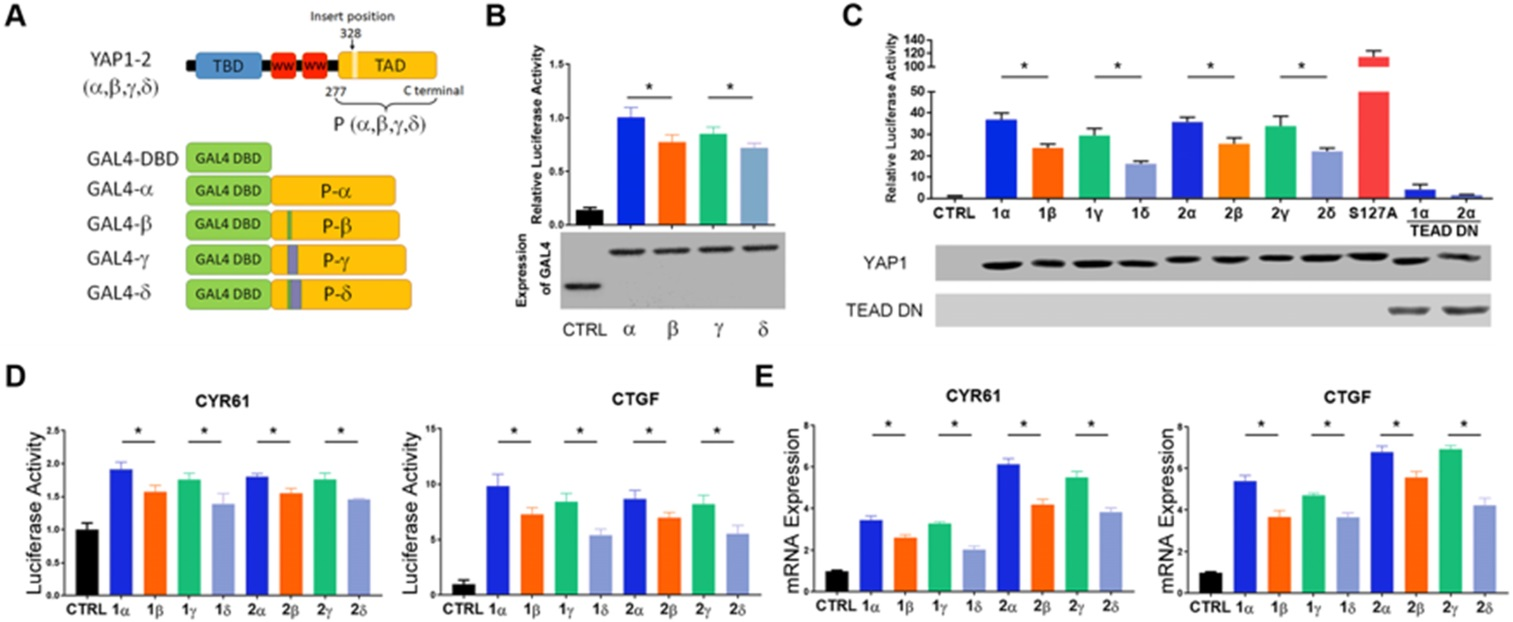
** Transcriptional activities of each YAP1 isoform. (A)** Schematic diagram of expression vectors carrying GAL4 DBD and YAP1(TAD) fused to DBD. TAD was amplified from cDNA encoding the 279^th^ a.a of YAP1-2 to the C terminus. **(B)** Relative activity of the GAL4 DBD control and GAL4 DBD-YAP1(TAD) luciferase reporters (*p<0.05). Note that the expression levels of the GAL4-YAP1(TAD) plasmids are comparable. **(C)** 8xGTIIC-luciferase reporter activity upon upon co-expression with each of YAP1 isoform or S127A mutant YAP1 in HEK293T cells. TEAD DN, expression plasmid encoding a.a. 101-426 of human TEAD1. **(D)** CYR61-promoter and CTGF-promoter reporter activity upon co-expression with indicated single YAP1 isoform in HEK293T cells. **(E)** qRT-PCR analyses of the mRNA expression of CYR61 and CTGF in L3.6-YAP1-x cells that express a single YAP1 isoform.

YAP1 harbors a potent TA domain located within the C-terminal region 25. The YAP1 α-isoforms contain a putative leucine zipper, which is interrupted at position 290 in YAP1-1 isoforms and at position 328 in YAP1-2 isoforms (Figure 2A). To determine the transcriptional activity of the TA domain of each of the four YAP1 isoforms, we first performed a GAL4-YAP1 (TA domain) luciferase reporter assay. All *GAL4-YAP1* showed robust activation of the heterologous luciferase reporter (Figure 2B). The activity of the YAP1 TA domain derived from the β and δ isoforms was significantly lower than that from the α and γ isoforms. However, no significant differences between α and γ or β and δ were observed. Since TEAD proteins are the predominant DNA-binding transcriptional partners of YAP1 26, we next determined the isoform-specific activity of full-length YAP1 on *8xGTIIC-luciferase,* a synthetic luciferase reporter containing 8 copies of TEAD-binding sites. The result was highly consistent with that of the *GAL4-luciferase* reporter and further indicated that the 2^nd^ WW domain has a minimal effect on transcriptional activity (Figure 2C). Serine 127 of YAP1 is a canonical phosphorylation site for LATS 1/2 leading to YAP1 cytoplasmic sequestration, whereas S127A mutation relieves this inhibition and promotes YAP1 nuclear translocation and transcriptional activity. As expected, the *YAP1-2α* (S127A) mutant displayed much higher transcriptional activity than its wild-type counterpart, whereas the coexpression of TEAD1 (a.a. 101-426) lacking the N-terminal DNA-binding domain functioned as a potent dominant-negative inhibitor of full-length YAP1 activity.

*CYR61* and *CTGF* are two well-known target genes of YAP1/TEAD complex 27, 28. Similar results were obtained upon examining the effect of each YAP1 isoform on the promoter activity of *CYR61* and *CTGF* in a dual luciferase assay (Figure 2D). This result was further validated for the endogenous mRNA expression of *CYR61* and *CTGF* in L3.6 cells reconstituted with each YAP1 isoform (Figure 2E). Overall, the results indicate that the insertion of 4 a.a. (VRPQ) in the β and δ isoforms negatively impacts the transcriptional activity of YAP1, whereas the insertion of 16 a.a. in γ and δ or the presence of the 2^nd^ WW in YAP1-2 has minimal influence in these assays.

### Cell density-dependent regulation of YAP1 isoform expression and localization

**Figure 3:**
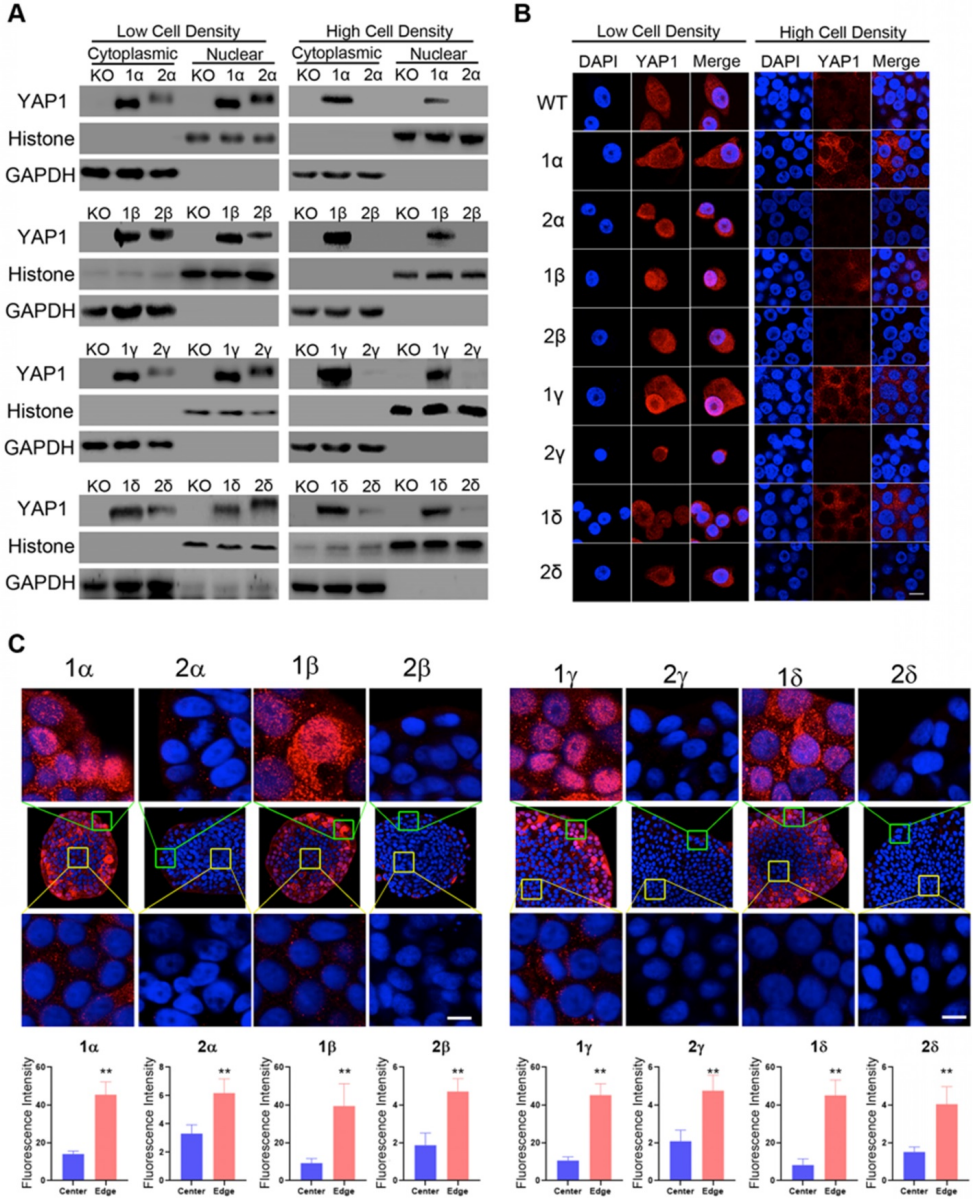
** Higher protein expression for YAP1-1 than YAP1-2 under high-density cell culture conditions. (A)** Western blot analysis of the cellular localization and expression of YAP1 isoforms in HCD and LCD conditions. **(B)** Immunofluorescence analysis of the cellular localization and expression of YAP1 isoforms in HCD and LCD conditions. Scale bar, 10μm. **(C)** Immunofluorescence analysis of the cellular localization and expression of YAP1 isoforms in monoclonal cells. The monoclonal cells were derived from cell culture in a plate seeded at an extremely low density growing for two weeks, Scale bar, 10μm.

To determine isoform specific response to cell density variations, we grew reconstituted KO-YAP1 L3.6 stable lines under high or low cell densities. Nuclear and cytoplasmic protein fractionation was performed to assess the distribution of YAP1 isoforms. In low cell density (LCD) cultures, all YAP1 isoforms were expressed at comparable levels and more abundantly detected in the nuclear fraction. However, under high cell density (HCD) conditions, the levels of YAP1-2 in the cytoplasm and the nuclei were drastically reduced compared to those of YAP1-1 (Figure 3A, Supplement Figure 3B). Given that the expression of reconstituted YAP1 in the L3.6-YAP1-x cells are almost the same in the subtypes of α, β, γ and δ (Figure 2D), we predicted that the differences in the protein levels of YAP1 isoforms should relate to post-transcriptional regulation.

Immunofluorescence staining for reconstituted YAP1 under LCD conditions showed the YAP1-1 and YAP1-2 isoforms were readily detected in both the cytosol and nuclei. However, under HCD conditions, YAP1-1 proteins were observed, albeit at reduced levels and more commonly in the cytoplasm, whereas YAP1-2 levels were dramatically reduced to barely detectable levels (Figure 3B), which is consistent with the immunoblot shown in Figure 3A.

Since cell contact is constantly changing during tumor proliferation and progression, as is likely the level and intracellular localization of YAP1, we next seeded cells at very LCD to derive single cell clones before immunofluorescence staining for YAP1. Interestingly, we found that YAP1-1 isoforms were abundantly expressed in the periphery of the cell colonies (Figure 3C) and tended to accumulate in the nuclei (Figure 3C top row), whereas they were greatly reduced and became mostly cytoplasmic in the center of the colony (Figure 3C bottom row). Strikingly, the overall levels of YAP1-2 expression were dramatically lower and, regardless of the region, mainly detected in the cytoplasm.

### YAP1-2 is more prone to ubiquitination-mediated degradation

Given that multiple YAP1 transcripts are expressed in PDAC cells, we next wanted to examine the expression of YAP1 proteins. Although no epitope-specific antibody is available to distinguish YAP1-1 from YAP1-2, YAP1-1 and YAP1-2 differ by 38 amino acids in the 2nd WW domain. As such even the longest YAP1-1 isoform, YAP1-1α (450 a.a), is 38 a.a. shorter than YAP1-2α (488 a.a.), making it possible to separate these protein isoforms by running longer gels (Figure 3A). We collected cell lysates from L3.6, Panc1 and BXPC3 cells for immunoblot analysis and used immunoprecipitated YAP1-1γ and YAP1-2γ as controls. Interestingly, the YAP1 bands from all three PDAC cell lines migrated closer to the size of YAP1-1γ, suggesting that endogenous YAP1-1 protein expression is higher than YAP1-2 protein expression, even though the levels of YAP1-2 mRNA are higher (Supplement Figure 3C).

**Figure 4:**
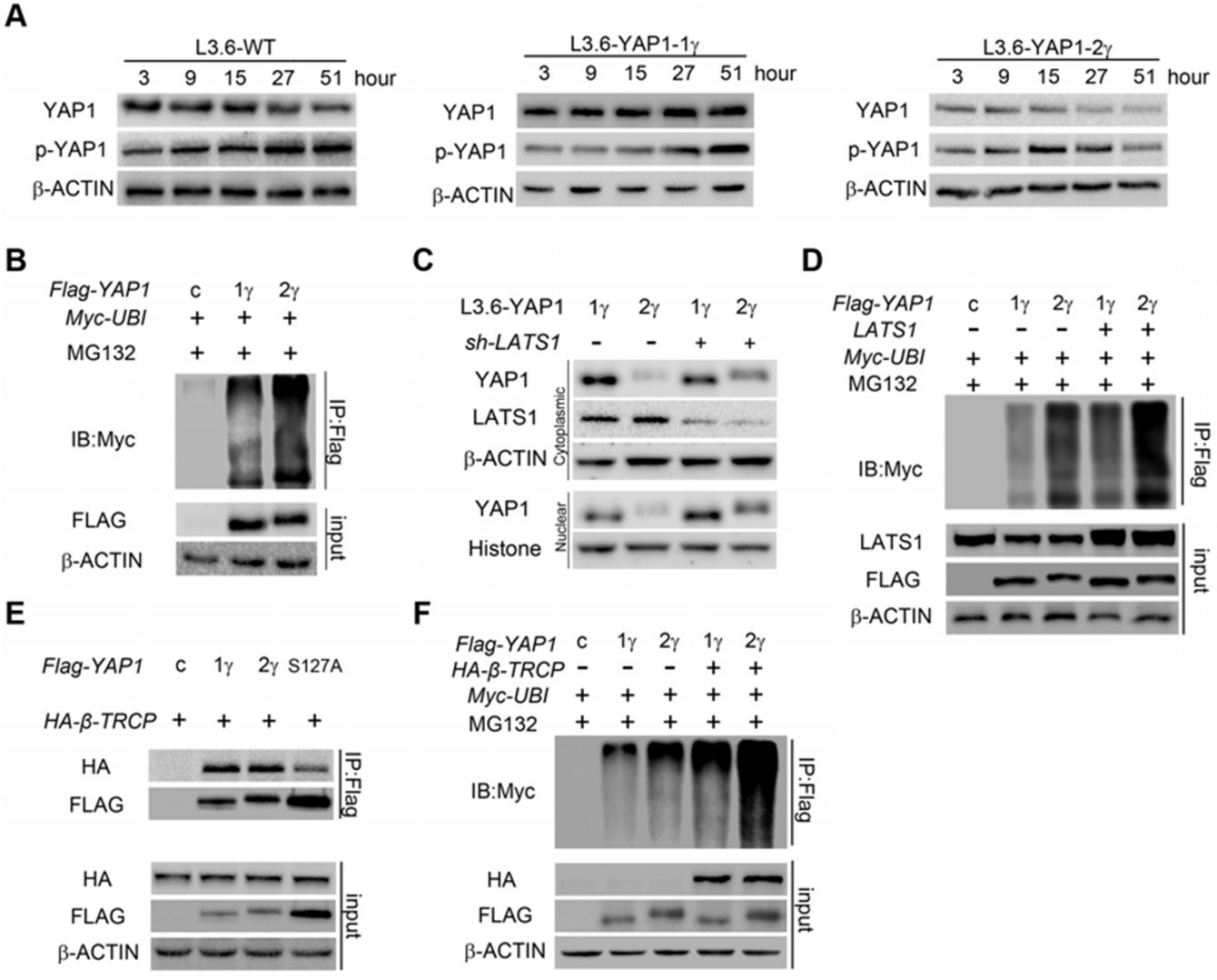
** YAP1-2 is more susceptible to ubiquitylation and degradation compared to YAP1-1. (A)** L3.6-YAP1-1γ and L3.6-YAP1-2γ cells were cultured in LCD conditions (10^6^ cells/10 cm dish) for 3 days to accumulate YAP1 proteins. The cells were then transferred to 3.5 cm dishes in HCD conditions (2×10^6^ cells/3.5 cm dish) to trigger degradation. Whole cell lysates of L3.6-YAP1-1γ and L3.6-YAP1-2γ cells were collected indicated time points and subjected to Western blotting to detect the abundance of YAP1 and p-YAP1. **(B)** Myc-tagged ubiquitin was co-transfected with either Flag-YAP1-1γ or YAP1-2γ into HEK293T cells as indicated. YAP1 ubiquitination was determined by IP for Flag and immunoblotting for myc. Transfection with *Flag-YFP* was used as a control. **(C)** L3.6-YAP1-1γ and L3.6-YAP1-2γ cells were cultured in HCD conditions, and lentiviruses containing *shYAP1* were added as indicated. Cytoplasmic and nuclear proteins were fractionated and subjected to Western blotting with indicated antibodies. **(D)** Myc-tagged ubiquitin and LATS1 were co-transfected with either *Flag-YAP1-1γ* or *YAP1-2γ* into HEK293T cells as indicated. YAP1 ubiquitination was determined by IP for Flag and immunoblotting for myc. Transfection with *Flag-YFP* was used as a control. **(E)** IP was used to detect the importance of YAP1-S127 in β-TRCP-mediated YAP1 ubiquitination. HA-tagged β-TRCP was co-transfected with Flag-tagged YAP1-1γ, YAP1-2γ or YAP1-2γ-S127A into HEK293T cells as indicated. The interaction of YAP1 and β-TRCP was determined by IP for Flag and immunoblotting for HA and YAP1. Transfection with Flag-YFP was used as a control. **(F)** IP was used to verify the function of β-TRCP in YAP1 ubiquitination. Flag-YAP1 (1γ or 2γ) and myc-tagged ubiquitin were co-transfected with or without β-TRCP. Transfection with Flag-YFP was used as a control. YAP1 ubiquitination was determined by IP for Flag and immunoblotting for myc.

The apparent discrepancy between YAP1-1 and YAP1-2 at mRNA and protein levels suggest a difference in protein stability. To test this, we chose L3.6-WT, L3.6-YAP1-1γ and L3.6-YAP1-2γ stable cell lines as representatives to investigate the degradation of the type 1 and type 2 isoforms. To accumulate enough protein to detect degradation, the cells were cultured at very LCD (10^6^ cells/10-cm dish) for 3 days and then trypsinized and replanted in 3.5 cm dishes at HCD (2×10^6^ cells/dish) for 3 h to adhere to the plate before sample collection for Western blot analysis (Figure 4A). The phosphorylation of YAP1 at S127 leads to its cytoplasmic retention followed by degradation 29. Although the phosphorylation level of the YAP1-1γ protein was increased at 27 h and peaked at 51 h, the total YAP1-1γ protein level did not decrease by 51 h. On the other hand, the level of the YAP1-2γ protein was markedly decreased starting at 27 h, whereas S127 phosphorylation was increased at 9 h, peaked at 15 h and then started decreasing. This result is consistent with YAP1-2 being more readily and robustly phosphorylated at S127, as we observed more rapid degradation of YAP1-2 than YAP1-1 at high cell densities. Ubiquitination assay was then carried out to determine whether YAP1-1 and YAP1-2 display different sensitivities to ubiquitin modification. We found that ubiquitination of YAP1-2γ was higher than that of YAP1-1γ (Figure 4B), suggesting that YAP1-2 is more susceptible to ubiquitination-mediated degradation at HCD.

LATS1 is a key kinase in the Hippo pathway activated upon cell adhesion leading to YAP1 phosphorylation at S127, cytoplasmic retention and degradation 30. We hypothesized that LATS1 may play a role in YAP1-2 degradation. We therefore knocked down LATS1 in L3.6-YAP1-1γ and L3.6-YAP1-2γ stable cells and determined YAP1 protein expression at HCD. The level of the YAP1-2 protein increased both in the cytoplasm and nucleus upon LATS1 suppression (Figure 4C). The ubiquitination of both YAP1-1γ and YAP1-2γ was further increased upon LATS1 overexpression, but the extent of the ubiquitination of YAP1-2γ was more robust than that of YAP1-1γ both under basal and LATS1 overexpression conditions (Figure 4D). These results together establish LATS1 as a critical factor in regulating YAP1-2 ubiquitination at high cell densities.

Several E3 ubiquitin ligases, including β-TRCP, are involved in regulating YAP1 ubiquitination and degradation. We found no obvious difference in the association of β-TRCP with YAP1-1γ or YAP1-2γ in the absence of MG132. However, compared to wild-type YAP1-2γ, the YAP1-2γ S127A mutant showed reduced binding to β-TRCP (Figure 4E). This result is consistent with the LATS1-mediated S127 phosphorylation leading to the ubiquitination and degradation of YAP1. Further experiments showed that, in the presence of MG132, YAP1-2 exhibited higher basal levels of ubiquitination, which was enhanced and more evident for YAP1-2 upon β-TRCP overexpression (Figure 4F). These results confirm that, in our cell model, β-TRCP is an E3 ubiquitin ligase for YAP1 and that the binding of β-TRCP and YAP1 is subject to regulation by LATS1. Our data also reveal higher stability of YAP1-1 than YAP1-2 at HCD due to its reduced ubiquitination, and LATS1 plays an important role in this process.

### Presence of the 2nd WW domain facilitates YAP1 binding to proteins with a PPxY motif

**Figure 5:**
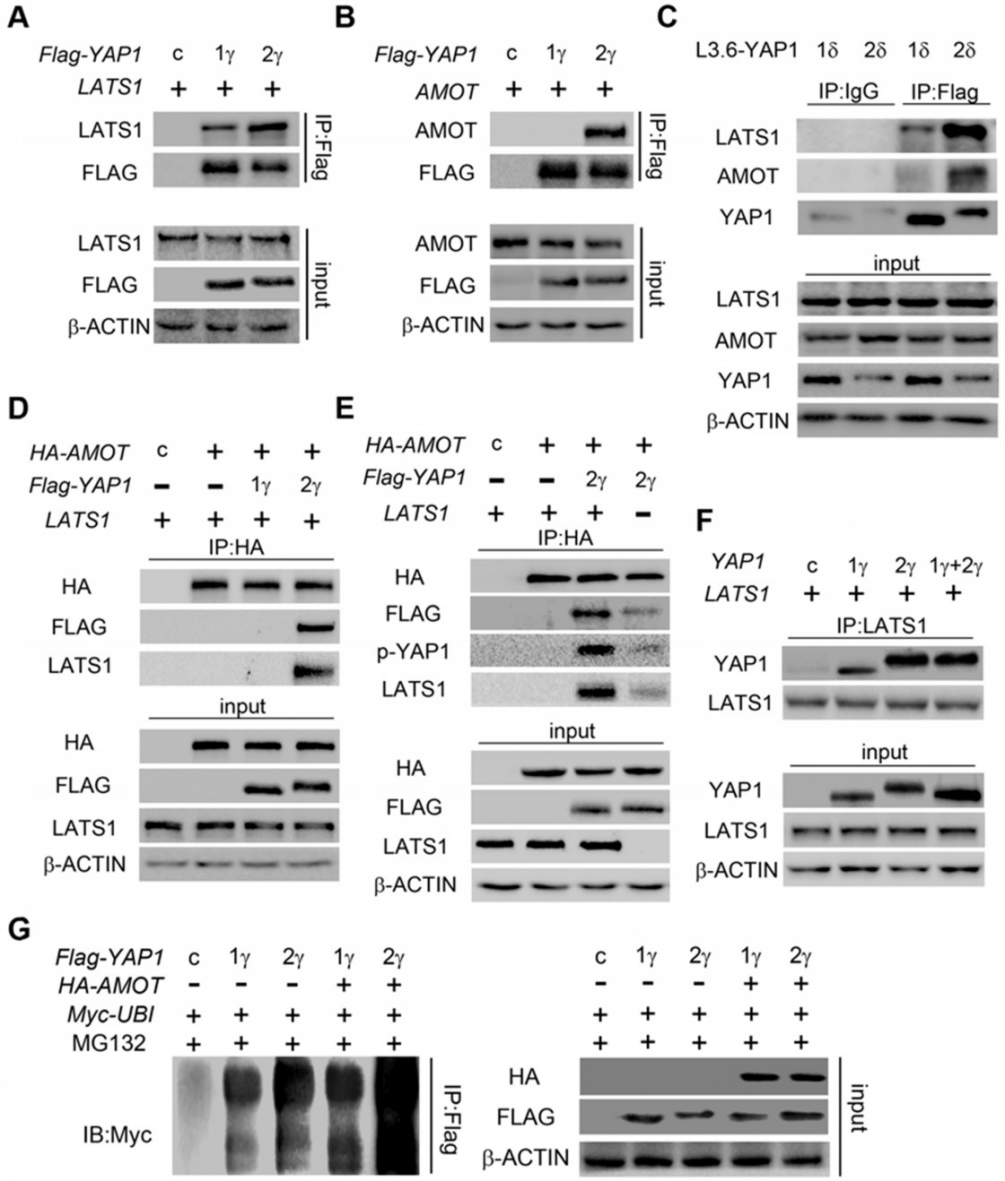
** YAP1-2, but not YAP1-1, can form a protein complex with both AMOT and LATS1.** *LATS1* **(A)**, *AMOT* **(B)**, were co-transfected with either *Flag-YAP1-1γ* or *Flag-YAP1-2γ* into HEK293T cells as indicated. The interaction of YAP1 and LATS1 or AMOT was determined by IP for Flag and immunoblotting for FLAG and either LATS1 or AMOT. Transfection with *Flag-YFP* was used as a control. **(C)** L3.6-YAP1-1δ and L3.6-YAP1-2δ cells were cultured in LCD conditions for 3 days to accumulate YAP1 protein. The cells were then plated in 10-cm dishes in HCD conditions and cultured for 24 h. The endogenous interaction of YAP1 with LATS1 or AMOT was determined by IP for Flag and immunoblotting for YAP1 and either LATS1 or AMOT. **(D)** *LATS1* and HA-tagged *AMOT* were co-transfected with either *Flag-YAP1-1γ* or *Flag-YAP1-2γ* into HEK293T cells as indicated. The interaction of AMOT, YAP1 and LATS1 was determined by IP for HA (AMOT) and immunoblotting for FLAG, LATS1 and HA. Transfection with *Flag-YFP* was used as a control. **(E)*** LATS1* and HA-tagged* AMOT* were co-transfected with *Flag-YAP1-2γ* into HEK293T cells as indicated. The interaction of AMOT, YAP1 and LATS1 was determined by IP for HA (AMOT) and immunoblotting for antibodies against Flag, p-YAP1, LATS1 and HA. Transfection with *Flag-YFP* was used as a control. **(F)** Co-IP analysis of the binding preference of LATS1 for YAP1-1 and YAP1-2. HEK293T cells were co-transfected with *LATS1* and *Flag-YAP1-1γ* and *Flag-YAP1-2γ* in the experimental group, with LATS1 in the control group, and with *Flag-YFP* as a negative control. **(G)** *Myc-tagged-ubiquitin* and *HA-tagged-AMOT* were co-transfected with either *Flag-YAP1-γ* or *YAPI-2γ* into HEK293T cells as indicated. YAP1 ubiquitation was determined by IP for Flag and immunoblotting for myc. Transfection with *Flag-YFP* was used as a control.

The WW domain mediates interactions of YAP1 with proteins that contain PPxY motifs, including LATS1/2^1^, AMOT^20^ and PTPN14^31^, ^32^. Many of these proteins are important YAP1-regulating proteins that can regulate the phosphorylation, nuclear localization, ubiquitination and degradation of YAP1. For clarification, we termed the common WW domain the 1^st^ WW domain and the additional WW domain of YAP1-2 proteins the 2^nd^ WW domain. We predicted that the 2^nd^ WW might impact interactions of YAP1 with PPxY motif containing proteins. Through co-IP analysis in HEK293T cells, we found that both YAP1-1γ and YAP1-2γ could bind LATS1, but that YAP1-2 displayed stronger binding than YAP1-1(Figure 5A), indicating that LATS1 can recognize the 1^st^ WW domain and that this interaction might somehow be strengthened by the 2^nd^ WW domain. AMOT and PTPN14 are two other PPxY motif-containing proteins of the Hippo pathway that also bind to and downregulate YAP1 31, 33. Indeed, we readily detected the binding of YAP1 to AMOT and PTPN14 in HEK293T cells, but found that only YAP1-2, but not YAP1-1, formed a complex with AMOT and PTPN14 under the same conditions (Figure 5B and Supplement Figure 3D). Similar results were obtained using L3.6-YAP1-1δ and L3.6-YAP1-2δ cells (Figure 5C and Supplement Figure 3E). Therefore, LATS1 binds to the 1^st^ WW domain of YAP1, and AMOT binds to the 2^nd^, which makes it highly possible for the three to form a complex. To verify this hypothesis, we performed co-IP to pull down AMOT after co-transfecting cells with LATS1, AMOT, and YAP1-1γ or YAP1-2γ. LATS1 was pulled down in the presence of YAP1-2γ but not YAP1-1γ (Figure 5D) and the binding of YAP1 to AMOT was enhanced by the overexpression of LATS1, accompanied by a high level of YAP1 phosphorylation, indicating that the interaction of AMOT and YAP1 relies on the activity of LATS1 (Figure 5E). AMOT is a membrane protein that mediates the degradation of YAP1. The LATS1-AMOT-YAP1-2 complex may therefore regulate YAP1 cytoplasmic retention and degradation.

The above findings led us to further hypothesize that, in the presence of both YAP1-1 and YAP1-2, LATS1 predominantly interacts with YAP1-2 and promotes its degradation. To verify this hypothesis, LATS1 was co-transfected with either control vector, YAP1-1γ, YAP1-2γ or YAP1-1γ and YAP1-2γ together. Western blotting of input cell lysates and LATS1 IP samples revealed that YAP1 co-IP with LATS1 matched to the YAP1-2γ band, but YAP1 of input samples matched mostly to the YAP1-1γ band (Figure 5F), indicating that LATS1 preferentially binds to YAP1-2. The ubiquitination of both YAP1-1γ and YAP1-2γ was further increased upon AMOT overexpression, but the extent of the ubiquitination of YAP1-2γ was greater than that of YAP1-1γ under both basal and AMOT overexpression conditions (Figure 5G).

### YAP1-1 has a stronger influence than YAP1-2 on cell malignancy

**Figure 6:**
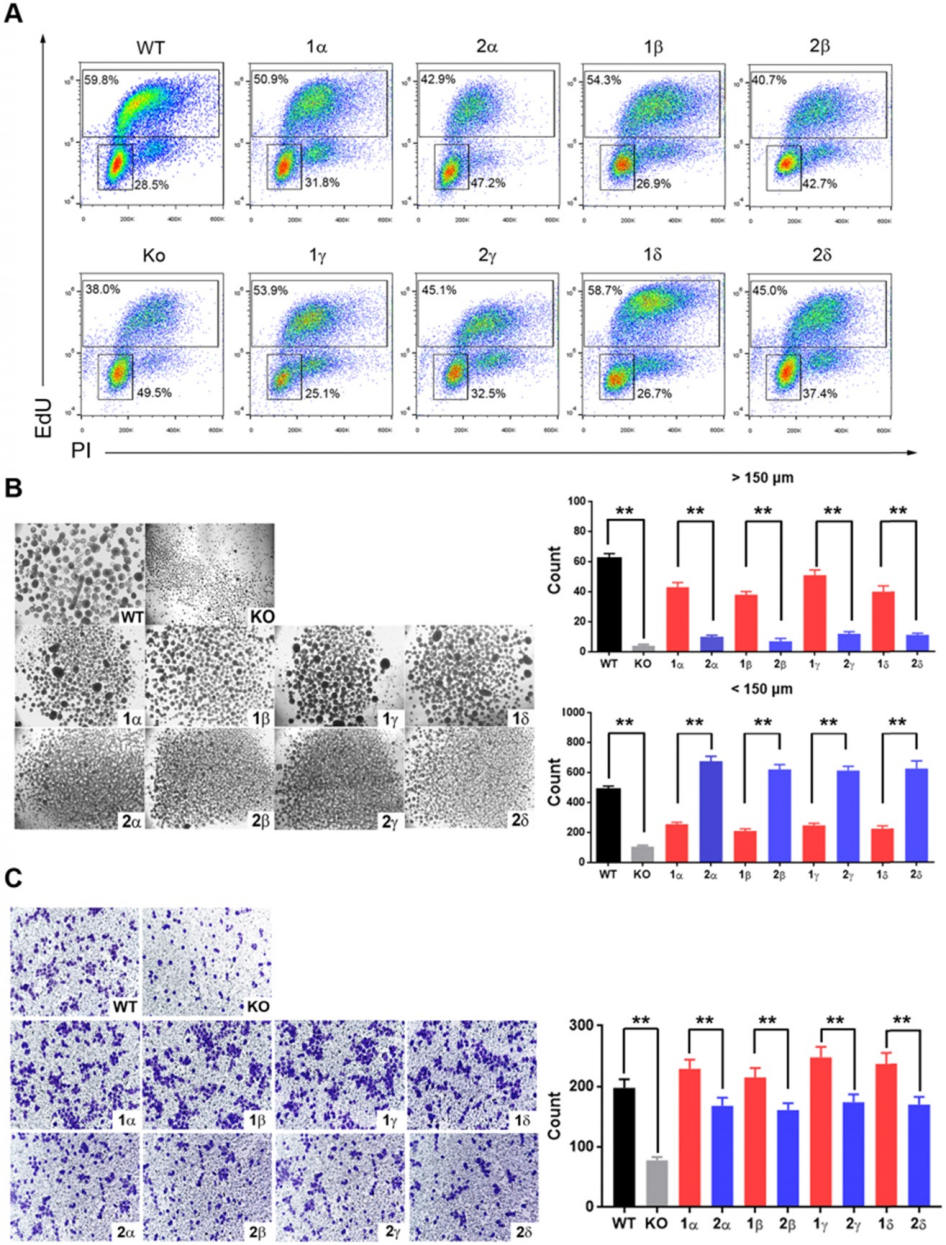
** YAP1-1 has a stronger influence than YAP1-2 on cell malignancy *in vitro*. (A)** EdU assay was used to analyze proliferation ability of L3.6-YAP1-x cells. **(B)** Sphere formation assay was carried out to access the stemness properties of L3.6-YAP1-x cells. The number of spheres >50 but <150 μm and the number of spheres >150 μm was counted for statistical analysis. **p<0.001. **(C)** Transwell assays were used to determine the migration ability of L3.6-YAP1-x cells. * p<0.05.

**Figure 7:**
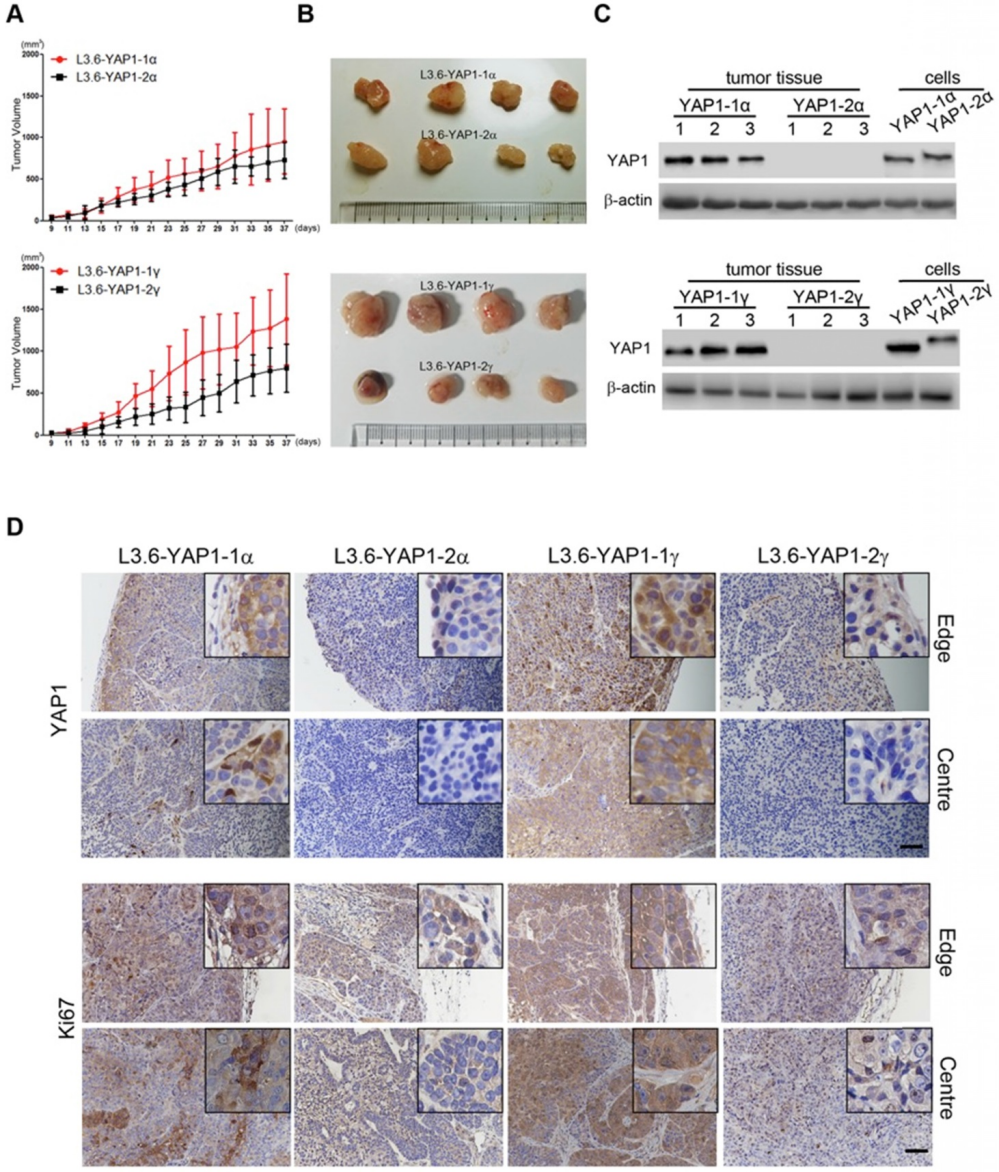
** YAP1-1 exerts stronger influence than YAP1-2 on cell proliferation *in vivo*.** BALB/c nu/nu mice were subcutaneously transplanted with 1 x 10^6^ of each L3.6-YAP1-x cells respectively (n=4). **(A)** The growth curves of tumor xenografts. The tumor volume was calculated by the formula: V= (length x width^2^)/2. **(B)** Representative macroscopic appearance of the tumors. **(C)** The expression of YAP1 in different types of tumors. **(D)** Tumor tissue sections were subjected to IHC analysis with YAP1 and Ki67 antibodies. Scale bar, 50μm.

YAP1 interacts predominantly with TEAD family to regulate the expression of downstream genes to promote tumorigenesis. The interaction of YAP1 with TEAD family members depends on its TAD, which is identical in YAP1-1 and YAP1-2. Therefore, there may be no difference in oncogenic functions of YAP1-1 and YAP1-2 when they are expressed at the same levels. As YAP1-1 protein displays higher stability, we hypothesized that YAP1-1 would exhibit stronger activity in promoting cell malignancy. We tested the proliferative and migratory capacity, as well as stem cell properties, of each L3.6-YAP1-x cell line *in vitro*. EdU assay was performed to determine the proliferation rate of each L3.6-YAP1-x cell line and L3.6-(KO-YAP1). The YAP1-1x cell lines showed a higher proportion of EdU-positive cells than the YAP1-2x cell lines (Figure 6A). MTT (Supplement Figure 4A) and colony formation (Supplement Figure 4B) assays further confirmed enhanced proliferation of the YAP1-1 cells. Sphere formation assay was performed to detect the stemness properties of each L3.6-YAP1-x cell line *in vitro*. L3.6-YAP1-1x cells formed a greater number of big spheres (>150 μm) than the L3.6-YAP1-2x cells. Contrastingly, the L3.6-YAP1-2x cells formed a greater number of small spheres (>50 but <150 μm) than L3.6-YAP1-1x cells (Figure.7B). Furthermore, wound healing (Supplement Figure 4C) and Transwell (Figure 6C) assays determined that L3.6-YAP1-1x cells exhibited a higher migratory capacity than the L3.6-YAP1-2x cells.

### YAP1-1 has a stronger influence than YAP1-2 on cell proliferation *in vivo*

**Figure 8:**
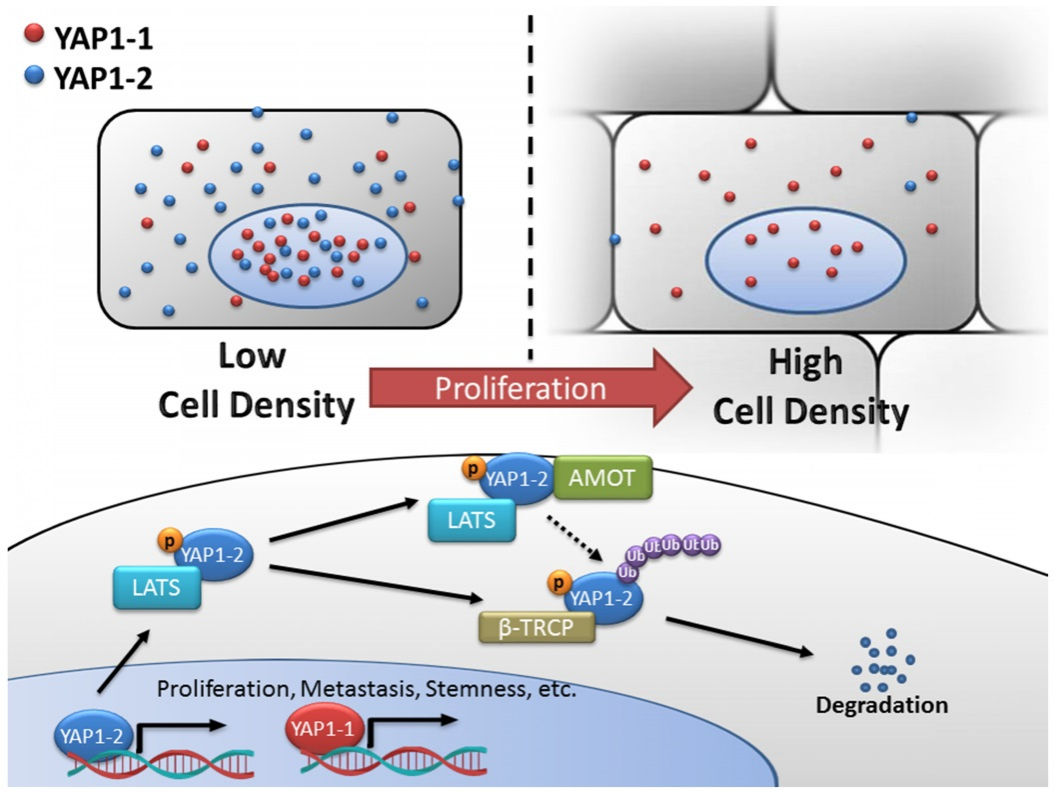
** Schematic diagram of the regulatory network of YAP1-1 and YAP1-2.** YAP1 expression and its intracellular localization are tightly regulated in response to various input signals including cell-cell contact. In the current study, systemic analysis and comparison of eight YAP1 isoforms were carried out and unveiled functional difference between the YAP1 subgroups and mechanism of differential regulation. At low density, both YAP1-1 and YAP1-2 protein isoforms can localize to nucleus to regulate target gene expression and promote proliferation, metastasis and stemness of cancer cells. However, at high cell density, activated Hippo pathway leads to YAP1 phosphorylation and cytoplasmic retention/sequestering. YAP1-2 with two WW domains is capable of forming de novo YAP1-2/LATS1/AMOT complex, rendering it more susceptible to LATS1/2 phosphorylation and subsequent degradation via β-TRCP-mediated ubiquitination.

Finally, we tested the tumorigenesis of each L3.6-YAP1-x cell line *in vivo*. L3.6-WT, L3.6-KoYAP1, L3.6-YAP1-1α, L3.6-YAP1-2α, L3.6-YAP1-1γ and L3.6-YAP1-2γ were chosen as representative cell lines for these experiments. The experimental cells were inoculated into the flanks of nude mice to generate xenografts and tumor growth was monitored on alternate days for 37 days. The primary tumors were collected for histological and pathological analysis at the time of sacrifice. Consistent with our *in vitro* data, the proliferation rates of the L3.6-YAP1-1α and L3.6-YAP1-1γ cell lines were higher than those of the L3.6-YAP1-2α and L3.6-YAP1-2γ cell lines (Figure 7A and B), whereas the proliferation and tumor growth rates of the L3.6-WT cells were higher than those of the L3.6-KoYAP1 cells (Supplement Figure 5A and B). Western blot analysis of tumor lysates revealed that YAP1 was barely expressed in the L3.6-YAP1-2α and L3.6-YAP1-2γ lines but was robustly expressed in the L3.6-YAP1-1α and L3.6-YAP-1γ lines (Figure 7C). Immunohistochemically staining showed consistently that YAP1 and Ki67 were expressed in the YAP1-1 lines, especially at the edge of the tumors. However, in the L3.6-YAP1-2α and L3.6-YAP1-2γ lines, their expression levels were much lower (Figure 7D). Consistent with reduced proliferation of L3.6-KoYAP1 tumor lines, their Ki67 expression levels were also much lower than L3.6-WT lines (Supplement Figure 5C). Thus, the proliferative capacity of YAP1-1 cells was stronger than that of the YAP1-2 cells *in vivo*, resulting in increased tumorigenic potential. The cartoon shown in Figure 8 illustrates our findings on the regulatory network of YAP1-1 and YAP1-2 (Figure 8).

## Discussion

The expression of YAP1 isoforms in human cancer including PDAC, and isoform-specific regulation and contribution to cancer initiation/ progression remain largely unexplored 15, 24. We show that multiple YAP1 mRNA isoforms are expressed in human PDAC cell lines, we uncover the discrepancies of mRNA and protein expression between YAP1-1 and YAP1-2, which prompted us to further address the regulation as well as the function of YAP1 protein isoforms in PDAC cells.

Many factors of the Hippo pathway such as LATS1/2, AMOT, and PTPN14, have PPxY motifs that can interact with the WW domain and regulate YAP1 signaling. It is not entirely surprising that having two WW domains allows YAP1-2 to interact more strongly and with multiple negative regulators. However, we found that the significance of WW-mediated interaction/degradation of YAP1 is exemplified under conditions of strong cell contact inhibition, for example, at the edges of clonal cells, where the concentration of YAP1-1 is much higher than that of YAP1-2 in both the cytoplasm and the nucleus. In solid tumor tissues, where cell contact inhibition dominates, although the mRNA level of YAP1-1 was much lower than that of YAP1-2, the protein levels exhibited the opposite pattern; thus, we suggest YAP1-1 plays a major role in tumorigenesis, whereas YAP1- 2, which become stabilized under low cell contact, may assume a more crucial role during EMT and tumor metastasis. It is believed that YAP1 interacts with TEAD family members via the TEAD binding domain in the N-terminus to accelerate cell malignancy 27, 34. However, this process is not related to the WW domain; thus, we speculate that YAP1-1 may play a more important role than YAP1-2 during the growth of solid tumors. This view is well supported by several cell malignancy studies including Sphere formation assay, which showed that the L3.6-YAP1-2x cells formed smaller albeit a greater number of spheres than the L3.6-YAP1-1x cells. One possible explanation is that, in cells possessing few cell-cell contacts, YAP1-2 stabilization in tumor cell contributes to maintain their stemness features. However, when the spheres grow larger, the cell-cell junctions become more abundant and YAP1-2 is degraded and loses its ability to promote the growth of the spheres.

Mechanistically, we show that AMOT and PTPN14 can only bind to YAP1-2, but not YAP1-1. Such differential binding is expected to occur with other partners of YAP1 and likely confer some new functional properties to YAP1-2 proteins. Considering that mRNA levels for YAP1-2 are much higher than YAP1-1 in cancer cells, the expression level of YAP1-2 protein may also be higher than that of YAP1-1 under low density cell culture conditions. Indeed, we observed that the expression levels of the YAP1 proteins in L3.6-YAP1-2 and L3.6-YAP1-1 cells are similar under LCD condition. Of note, this result was obtained in singe isoform reconstituted expressing cell lines, meaning that the mRNA of YAP1-1 and YAP1-2 are expressed at similar levels. Additionally, we found that when LATS1 is overexpressed, total YAP1 and S127-phosphorylated YAP1*,* which bind to AMOT, are increased, indicating that YAP1 might be phosphorylated by LATS1 before interacting with AMOT. It has been reported that the overexpression of AMOT can lead to the degradation of YAP1 and vice versa 33. Therefore, the LATS1-YAP1-2-AMOT complex may be involved in the degradation of both YAP1-2 and AMOT through LATS1.

Our findings together indicate that the 2^nd^ WW domain not only in conferring stronger binding to some PPxY motif containing proteins such as LATS1, but also different binding preferences including de novo protein complex formation such as* LATS1-YAP1-2-AMOT,* which might contribute to modulating target recognition between the two YAP isoforms^35^-^37^. Given that the WW domain not only interacts with the negative cytosolic regulators mentioned above, but also nuclear factors such as ZEB1, RUNX, P73, and SMAD, which may contribute to a different spectrum of target gene regulation and biological function. Active escaping from the primary site to enter blood stream as circulating tumor cells (CTCs) is a prerequisite step for distant metastasis of solid tumors. CTCs do not subject to cell contact inhibition; thus, we assume that YAP1-2 may be abundantly expressed at this stage. YAP1 has always been considered a factor that can promote cell proliferation through interaction with the TEAD family. However, in CTCs, when proliferation is not the major task of cancer cells, YAP1 may interact with other factors and regulate other functions, for example survival against anoikis. If this unknown pathway exists, it is highly possible that it involves YAP1-2 and that the target genes of YAP1 may be different in CTCs from that of solid tumors. In light of this, we suggest that it might be more appropriate to use YAP1-2 to study the YAP1 function in CTCs, while YAP1-1 should be used to study YAP1 functions in cell proliferation and tumorigenesis.

In summary, the current study, to our knowledge for the first time, systemically characterize the expression, regulation and function of YAP1 isoforms. Together, our work indicates that multiple YAP1 mRNA isoforms are simultaneously expressed in human PDAC cell lines in a highly regulated manner, particularly at protein level in response to cell density for which the 2^nd^ WW domain plays a pivotal role. Mechanistically, we unveil that the 2nd WW motif not only enhances YAP1 interaction with LATS1 leading to its ubiquitination and degradation, but also mediates LATS1/YAP1-2/AMOT complex formation. While YAP1-1 is more potent than YAP1-2 in promoting cancer cell malignancy in culture and primary tumor growth *in vivo*, YAP1-2 stabilized under low cell contact/density such as in CTCs, may contribute to cancer metastasis that merits future investigation. Our findings on the differential expression, regulation and function of YAP1 protein isoforms provide insight on the role of Hippo/YAP1 signaling in tumorigenesis and targeting of YAP1 for cancer therapy.

## Materials and Methods

### Plasmid and cDNA cloning

We* used YAP1-2α* (ORF=488 a.a.) from *pCMV-2xFlag-YAP1* (Addgene #19045) as template and overlapping PCR to generate all full-length YAP1-1 and YAP1-2 isoforms (Figure 1C)*,* which were further subcloned into a *pLenti6.3 vector* (Invitrogen, USA) for stable expression.

The cDNAs encoding the C-termini of four *YAP1* TA domain isoforms were cloned into the *pM* vector for Gal4-reporter assay*.* The cDNA *encoding* a.a. 101-426 of *TEAD1* was cloned *and used as* a dominant-negative version of full-length. The *pLKO.1* vector was used to construct LATS1 and LATS2 shRNA lentiviral expression vectors. YAP1 knockout vector was constructed in *pSpCas9 (BB)-2A-Puro* (Addgene #62988). TEAD reporter plasmid (*8xGTIIC- luciferase*) was obtained from Addgene (#34615). The *CTGF* and *CYR61* promoter luciferase reporter plasmids were kind gifts from Thomas Brabletz (FAU University Erlangen-Nurnberg). Other plasmids, including *pCMV2.2xFlag-YAP2 (127A), pcDNA3.1-HA- LATS1, pCMV-HA-TRCP, pcDNA3-HA-AMOT (p130), pcDNA3-V5-PTPN14,* and Myc or HA tagged ubiquitin expression vectors were obtained from Addgene or cloned using standard molecular biology techniques. Please refer to Supplementary materials and Methods for details of YAP1 gene knockout and isoform expression plasmids.

### Cell culture, transfection and the generation of the YAP1 knockout cell line

HEK293T and the PDAC lines L3.6, BxPC3, and PANC-1 were maintained and transfected as previously described 38. To generate YAP1 knockout cells, *YAP1-sgRNA-PX459* and 1/5 of the *GFP* expression plasmid were transiently transfected into L3.6 cells using Lipofectamine 3000. GFP-positive cells were sorted into a 96-well plate 24 h post transfection at 1 cell/well using a BD Biosciences FACSAria II cell sorter and allowed to grow*.* A single cell-derived clone (KO-YAP1) was validated for null *YAP1* expression by Western blotting.

### Lentiviral packaging, viral transduction and selection of stable cells

Lentiviral packaging, cell infection and *pLKO-shRNA* stable cell selection were performed as previously described 38. For the stable reconstituted expression of YAP1-isoforms, lentiviral particles carrying *pLenti6.3-Flag-YAP1* cDNA encoding each YAP1-specific isoform were used to infect *L3.6 KO-YAP1* cells. The cells were then selected with blasticidin (5 μg/mL), and the pooled resistant cells were used as stable overexpression cells.

### RNA isolation, real-time PCR and YAP1 isoform detection

Total RNA was extracted with RNAiso Plus (TaKaRa). The PrimeScript RT Reagent Kit (TaKaRa) was used for cDNA synthesis. Real-time PCR was carried out with the CFX96 Real-Time System (Bio-Rad) and SYBR Premix Ex Taq (TaKaRa). YAP1 specific primers were designed to amplify the cDNA fragment flanking the 2^nd^ WW and the region of the TA domain subjected to alternative splicing. Purified RT-PCR products were cloned into the pGEM-T vector. The sequencing results were compiled and calculate the proportion of each isoform relative to the total. The detailed information of primers, amplicons and expression analysis can be found in Supplementary materials and Methods.

### Luciferase reporter assays

GAL4-luciferase reporter assays were performed as previously described, with minor modifications 38. Briefly, HEK293T cells were transfected with a *pGL3* firefly reporter plasmid containing five tandem GAL4 DNA binding sites (*GAL4-luc*) and a *pM-GAL4-YAP1* (TA-domain) effector plasmid. Luciferase assays were performed with the GloMax® Navigator System (Promega). To test the transcriptional activity of YAP1 on its downstream target genes, HEK293T cells were transfected with *pGL4.1* firefly reporter plasmid carrying *CTGF* or *CYR61* and the *YAP1* isoform-expressing plasmids. The results were normalized to renilla luciferase activity and are expressed as the mean fold induction. Mean values of at least three independent experiments are displayed as the mean±S.D.

### Immunofluorescent staining and imaging

Cells were grown on glass bottom cell culture dishes (NEST, 801002). For LCD and HCD cultures, 100 cells or 3×10^4^ cells, respectively, were plated per dish and cultured for 24 h before staining. For monoclonal cell culture, 30 cells/dish were plated and cultured for 2 weeks. Immunofluorescence staining was carried out with YAP1 (CST, D8H1X) as primary antibody, donkey anti-rabbit Alexa Fluor® 647 (Abcam, ab150075) as the secondary, and DAPI (D1306, Thermo Fisher) for counterstaining. Confocal images were obtained with Leica SP8 confocal microscope and Suite-Advanced Fluorescent software.

### Immunoprecipitation and immunoblotting

Whole cell lysates were prepared in RIPA buffer supplemented with a protease inhibitor cocktail (Roche, Basel, Switzerland) and quantified using the Bradford assay (LEAGENE, PT0010). Protein extracts (500 μg) were incubated with the appropriate antibody beads overnight. The precipitated immune complexes were subjected to SDS-PAGE and immunoblotted with antibodies against YAP1 (CST, D8H1X), phospho-YAP1 (Serine 127, CST, D9W2I), LATS1 (CST, C66B5), AMOT (Proteintech, 24550-1-AP), PTPN14 (CST, D5T6Y), β-actin (Sigma, A2228), histone (Beyotime, AF0009), Flag (CST, D6W5B), HA (Sigma, H3663) and myc (CST, 2272). Immunoreactive bands were detected using the ChemiDoc™ XRS+ System (Bio-Rad).

### Ubiquitination assay

HEK293T cells were transfected with the *Flag-YAP1* and *Myc-UbI* vectors and incubated with 20 μM MG132 for 4 h before harvesting. Cells were washed twice with prechilled PBS and lysed in 120 μL buffer with 10% glycerol, 1% SDS, pH 6.8 Tris-HCl (62.5 mM), 1 mM iodoacetamide and 10 mM NEM. Cell lysates were boiled for 15 min and then diluted with NTEN lysis buffer freshly supplemented with protease and deubiquitination inhibitors at a 1:9 ratio. The cell lysates were immunoprecipitated with anti- flag M2 agarose beads and the immune complexes were subjected to Western blotting.

### Cell proliferation assay

Cell proliferation was determined by MTT, EdU incorporation, and colony formation assays, respectively. MTT were performed from days 1 to 10 after plating using the Cell Proliferation Kit I (Sigma). For EdU assay, cells were cultured in serum-free medium for 24 h, and then replaced with complete medium. Cell proliferation was assessed using the Cell-Light EdU Apollo488 *in vitro* Flow Cytometry Kit (RiboBio) according to the manufacturer's instructions. The EdU-positive cells were viewed with the ACCURI C6 system (BD).For colony formation assay, a total of 1000 cells were plated in 6-well plates and allowed to grow until visible colonies formed (about 14 days). The cells were fixed with 100% methanol for 10 min at room temperature and stained with 0.5% crystal violet for 20 min at room temperature.

### Sphere formation assay

L3.6-YAP1-x cells were resuspended in standard stem cell medium (SCM), which consisted of the following: DMEM/F12 (Invitrogen; Thermo Fisher Scientific, Inc.) supplemented with 1X B27 (Invitrogen; Thermo Fisher Scientific, Inc.), 20 ng/mL human recombinant epidermal growth factor (Sigma-Aldrich; Merck KGaA) and 20 ng/mL FGF2 (Sigma-Aldrich; Merck KGaA). The spheres were cultured at 37°C in humidified air containing 5% CO_2_. To calculate sphere formation efficiency, 7 days after plating, spheres with a diameter >50 µm but <150 µm and those with a diameter >150 µm were counted under an inverted microscope.

### Transwell assay

The cell migration assay was performed using a Boyden chamber in a 24-well plate designed by Cell Biolabs Inc. (San Diego, CA, United States). Briefly, for each condition, cells were suspended at 10^6^ Cells per mL in serum-free RPMI1640 medium and added 200 mL to the upper chamber of each well. The same medium supplemented with 10% serum was added to the lower chamber as a chemoattractant. After 24 h, the cells that migrated to the lower chamber of each well were stained using a crystal violet cell staining solution. The stained cells were counted for statistical analysis.

### Mouse xenograft model

Male athymic nude mice on a BALB/c background were purchased from Laboratory Animal Center of Wenzhou Medical University. The mice were housed and maintained in laminar flow cabinets under specific pathogen-free conditions. The mice (8 to 12 weeks old) were used in accordance with institutional guidelines.

For *in vivo* injections, cells were harvested and suspended in serum-free Hanks' balanced salt solution (HBSS). Single-cell suspensions of greater than 95% viability, based on trypan blue exclusion, were used for injection. Each experimental group contained 6 animals. A total of 10^6^ tumor cells in 200 μl of HBSS were injected into the flank of each mouse. From day 9 post-injection, tumor progression was monitored by palpation and, every other day, caliper measurements along the longest and shortest diameters of the tumor were performed. The tumor volume was calculated with an established formula (Volume = [(length) x (width)^2^]/2). The mice were sacrificed 37 days post-transplantation. Fresh isolated tumor tissues were arranged in rows for imaging before being fixed with formalin. The sections were processed for immunohistochemical staining with primary antibodies against (CST, D8H1X at 1:200) and Ki67 (Abcam, ab15580).

### Statistical analysis

Luciferase reporter assays, real-time PCR, MTT assay and tumor growth curves are presented as the mean±S.D. *p* values showing differences were calculated by an unpaired two-tailed t test, and those showing no differences were calculated by a one-tailed t test.

## Supplementary Material

Supplementary figures and methods.
