# The WW domains dictate isoform-specific regulation of YAP1 stability and pancreatic cancer cell malignancy: Erratum

**DOI:** 10.7150/thno.51728

**Published:** 2020-08-18

**Authors:** Qiang Guo, Meiyu Quan, Jinglai Dong, Jing Bai, Jie Wang, Rui Han, Wei Wang, Yaxin Cai, Yu-Qing Lv, Qianjie Chen, Huijing Xu, Han-Deng Lyu, Liancheng Deng, Depu Zhou, Xueyuan Xiao, Stijn De Langhe, Daniel D. Billadeau, Zhenkun Lou, Jin-San Zhang

**Affiliations:** 1 School of Pharmaceutical Sciences, Wenzhou Medical University, Wenzhou, Zhejiang 325035, China; 2 Center for Precision Medicine, the First Affiliated Hospital of Wenzhou Medical University, Wenzhou, Zhejiang 325000, China; 3 Key Laboratory of Cell Proliferation and Regulation Biology, Ministry of Education, Beijing Normal University, Beijing 100875, China; 4 Department of Medicine, Division of Pulmonary, Allergy and Critical Care Medicine, University of Alabama at Birmingham, Birmingham, 35294-2182 AL, USA; 5 Division of Oncology Research, and Schulze Center for Novel Therapeutics, Mayo Clinic, Rochester, MN 55905, USA; 6 Institute of Life Sciences, Wenzhou University, Wenzhou, Zhejiang 325035, China

We noticed some errors in the initially published version of this article on the control immunoblot results shown in Figure 4E and Figure 5A, D, E, F, as well as transwell assay in Figure 6C. The figures with those errors corrected are shown below. The corrections made in this erratum do not affect the original conclusions or any part of the text and figure legends. The authors wish to apologize for any inconvenience or misunderstanding that these errors may have caused.

## Figures and Tables

**Figure 4:**
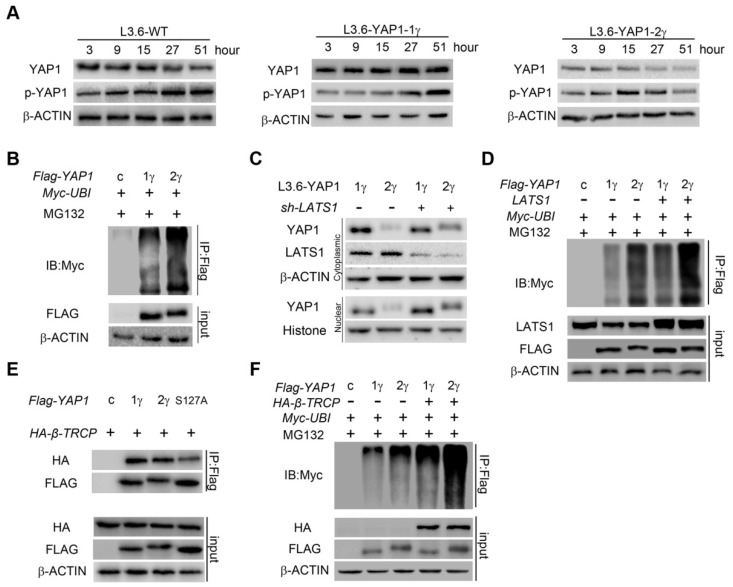
** YAP1-2 is more susceptible to ubiquitylation and degradation compared to YAP1-1. (A)** L3.6-YAP1-1γ and L3.6-YAP1-2γ cells were cultured in LCD conditions (10^6^ cells/10 cm dish) for 3 days to accumulate YAP1 proteins. The cells were then transferred to 3.5 cm dishes in HCD conditions (2×10^6^ cells/3.5 cm dish) to trigger degradation. Whole cell lysates of L3.6-YAP1-1γ and L3.6-YAP1-2γ cells were collected indicated time points and subjected to Western blotting to detect the abundance of YAP1 and p-YAP1. **(B)** Myc-tagged ubiquitin was co-transfected with either Flag-YAP1-1γ or YAP1-2γ into HEK293T cells as indicated. YAP1 ubiquitination was determined by IP for Flag and immunoblotting for myc. Transfection with *Flag-YFP* was used as a control. **(C)** L3.6-YAP1-1γ and L3.6-YAP1-2γ cells were cultured in HCD conditions, and lentiviruses containing *shYAP1* were added as indicated. Cytoplasmic and nuclear proteins were fractionated and subjected to Western blotting with indicated antibodies. **(D)** Myc-tagged ubiquitin and LATS1 were co-transfected with either *Flag-YAP1-1γ* or *YAP1-2γ* into HEK293T cells as indicated. YAP1 ubiquitination was determined by IP for Flag and immunoblotting for myc. Transfection with *Flag-YFP* was used as a control. **(E)** IP was used to detect the importance of YAP1-S127 in β-TRCP-mediated YAP1 ubiquitination. HA-tagged β-TRCP was co-transfected with Flag-tagged YAP1-1γ, YAP1-2γ or YAP1-2γ-S127A into HEK293T cells as indicated. The interaction of YAP1 and β-TRCP was determined by IP for Flag and immunoblotting for HA and YAP1. Transfection with Flag-YFP was used as a control. **(F)** IP was used to verify the function of β-TRCP in YAP1 ubiquitination. Flag-YAP1 (1γ or 2γ) and myc-tagged ubiquitin were co-transfected with or without β-TRCP. Transfection with Flag-YFP was used as a control. YAP1 ubiquitination was determined by IP for Flag and immunoblotting for myc.

**Figure 5:**
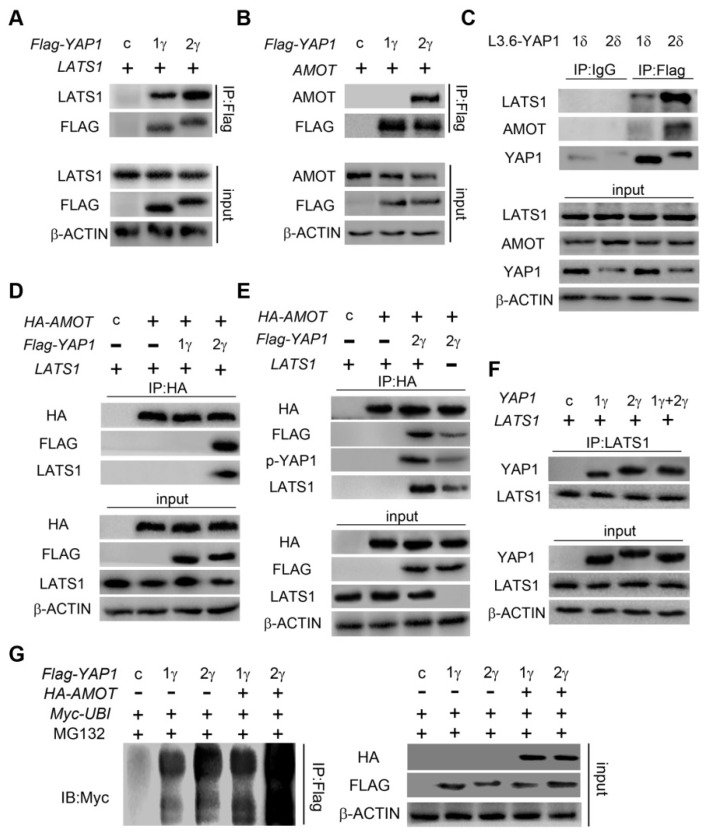
** YAP1-2, but not YAP1-1, can form a protein complex with both AMOT and LATS1.** *LATS1* **(A)**, *AMOT* **(B)**, were co-transfected with either *Flag-YAP1-1γ* or *Flag-YAP1-2γ* into HEK293T cells as indicated. The interaction of YAP1 and LATS1 or AMOT was determined by IP for Flag and immunoblotting for FLAG and either LATS1 or AMOT. Transfection with *Flag-YFP* was used as a control. **(C)** L3.6-YAP1-1δ and L3.6-YAP1-2δ cells were cultured in LCD conditions for 3 days to accumulate YAP1 protein. The cells were then plated in 10-cm dishes in HCD conditions and cultured for 24 h. The endogenous interaction of YAP1 with LATS1 or AMOT was determined by IP for Flag and immunoblotting for YAP1 and either LATS1 or AMOT. **(D)** *LATS1* and HA-tagged *AMOT* were co-transfected with either *Flag-YAP1-1γ* or *Flag-YAP1-2γ* into HEK293T cells as indicated. The interaction of AMOT, YAP1 and LATS1 was determined by IP for HA (AMOT) and immunoblotting for FLAG, LATS1 and HA. Transfection with *Flag-YFP* was used as a control. **(E)*** LATS1* and HA-tagged* AMOT* were co-transfected with *Flag-YAP1-2γ* into HEK293T cells as indicated. The interaction of AMOT, YAP1 and LATS1 was determined by IP for HA (AMOT) and immunoblotting for antibodies against Flag, p-YAP1, LATS1 and HA. Transfection with *Flag-YFP* was used as a control. **(F)** Co-IP analysis of the binding preference of LATS1 for YAP1-1 and YAP1-2. HEK293T cells were co-transfected with *LATS1* and *Flag-YAP1-1γ* and *Flag-YAP1-2γ* in the experimental group, with LATS1 in the control group, and with *Flag-YFP* as a negative control. **(G)** *Myc-tagged-ubiquitin* and *HA-tagged-AMOT* were co-transfected with either *Flag-YAP1-γ* or *YAPI-2γ* into HEK293T cells as indicated. YAP1 ubiquitation was determined by IP for Flag and immunoblotting for myc. Transfection with *Flag-YFP* was used as a control.

**Figure 6:**
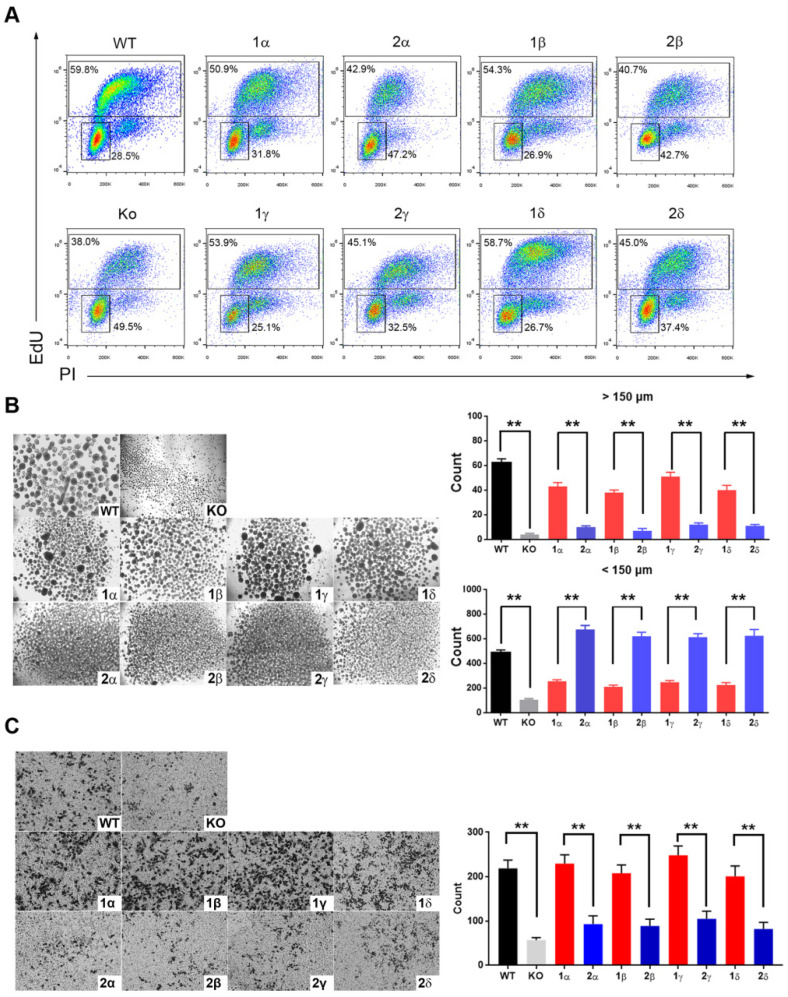
** YAP1-1 has a stronger influence than YAP1-2 on cell malignancy *in vitro*. (A)** EdU assay was used to analyze proliferation ability of L3.6-YAP1-x cells. **(B)** Sphere formation assay was carried out to access the stemness properties of L3.6-YAP1-x cells. The number of spheres >50 but <150 μm and the number of spheres >150 μm was counted for statistical analysis. **p<0.001. **(C)** Transwell assays were used to determine the migration ability of L3.6-YAP1-x cells. * p<0.05.

