# Supplementary material for: The WW domains dictate isoform-specific regulation of YAP1 stability and pancreatic cancer cell malignancy
Source: Theranostics. 2020 Mar 15;10(10):4422–36. doi: 10.7150/thno.42795 (PMC7150473; doi:10.7150/thno.42795)
Supplement: Supplementary file 1 — Supplementary figures and methods. [file thnov10p4422s1.pdf]

SUPPLEMENTARY FIGURES, MATERIALS AND METHODS

Supplement Figure 1

```
        10         20         30         40         50
MDPGQQPPPQ PAPQGQGQPP SQPPQGQGPP SGPGQPAPAA TQAAPQAPPA
        60         70         80         90        100
GHQIVHVRGD SETDLEALFN AVMNPKTANV PQTVPMRLRK LPDSFFKPPE
       110        120        130        140        150
PKSHSRQAST DAGTAGALTP QHVRAHSSPA SLQLGAVSPG TLTPTGVVSG
       160        170        180        190        200
PAATPTAQHL RQSSFEIPDD VPLPAGWEMA KTSSGQRYFL NHIDQTTTWQ
       210        220        230        240        250
DPRKAMLSQM NVTAPTSPPV QQNMMNSASG PLPDGWEQAM TQDGEIYYIN
       260        270        280        290        300
HKNKTTSWLD PRLDPRFAMN QRISQSAPVK QPPPLAPQSP QGGVMGGSNS
       310        320        330        340        350
NQQQQMRLQQ LQMEKERLRL KQQELLRQVR PQAMRNINPST ANSPKCQEL
       360        370        380        390        400
ALRSQLPTLE QDGGTQNPVS SPGMSQELRT MTTNSSDPFLN SGTYHSRDE
       410        420        430        440        450
STDSGLSMSS YSVPRTPDDF LNSVDEMDTG DTINQSTLPS QQNRFPDYLE
       460        470        480        490        500
AIPGTNVDLG TLEGDGMNIE GEELMPSLQE ALSSDILNDM ESVLAATKLD
       510
KESFLTWL
```

S LATS1/2 phosphorylation site

The first WW domain
VPLPAGWEMAKTSSGQRYFLNHIDQTTTWQDPRK

The second WW domain
GPLPDGWEQAMTQDGEIYYINHKNKTTSWLDPRL

The amino acid sequence distinguishes YAP1-1 from YAP1-2
GPLPDGWEQAMTQDGEIYYINHKNKTTSWLDPRLDPRF

The amino acid sequence distinguishes α, β, γ and δ
VRPQAMRNINPSTANSPKCQ

The transcriptional activation domain (TAD)
QGGVMG......SFLTWL

Diagram of YAP1 amino acid sequence and annotated functional domains and site.

Supplement Figure 2

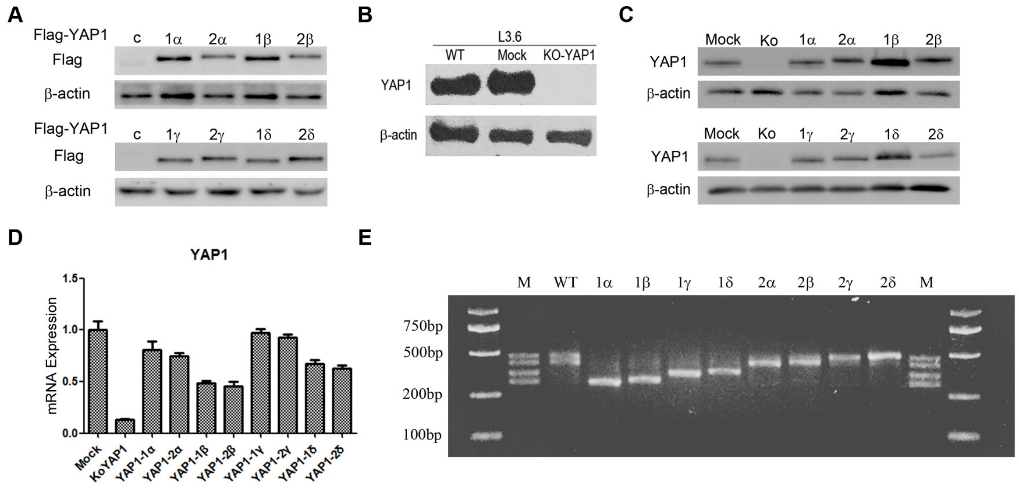

Validation of YAP1 overexpression, knockout and reconstituted single isoform expression: (A) Western blot analysis of Flag tagged *YAP1* constructs transfected in HEK293 cells. (B) Western blot analysis to validate the YAP1 knockout in L3.6 cell line. (C) Western blot analysis of the L3.6-YAP1-x cell lines derived from the YAP1 knockout cell line reconstituted with specified single YAP1 isoform. (D) qRT-PCR to validate the expression of YAP1 in L3.6-YAP1-x cell lines. (E) Reverse transcriptional PCR to validate the isoform-specific *YAP1* amplicons in L3.6-YAP1-x cell lines. The products were amplified from cDNA of the indicated stable cell lines and a wild-type L3.6 cell line using *YAP1*-specific primers. The M lane represents the mixture of products amplified from the YAP1 single isoform-expressing plasmids.

Supplement Figure 3

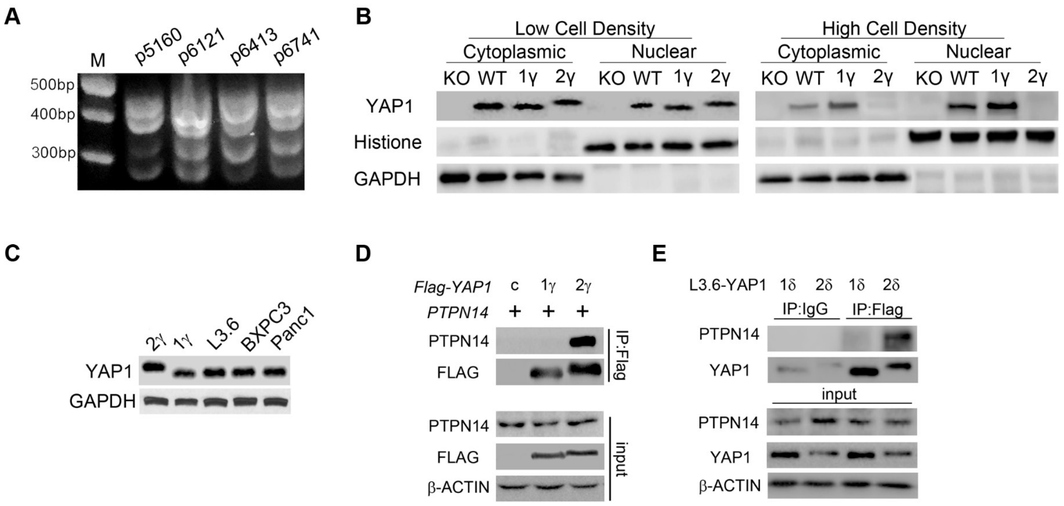

Expression of multiple YAP1 mRNA isoforms in PDAC/PDX cell lines, differential expression/localization of YAP1 protein isoforms and their interactions with PTPN14: (A) PCR products amplified from the cDNA of human pancreatic cancer PDX cells. (B) Western blot analysis of the fractionated protein samples for cellular localization and expression of YAP1 isoforms under HCD and LCD conditions. (C) Western blot analysis of the endogenous YAP1 protein isoforms in wild type L3.6, Panc1 and BXPC3 cells. L3.6-YAP1-1γ and L3.6-1-2γ transfected cell lysates were used as reference for YAP1-1 and YAP1-2 proteins. (D) PTPN14 was co-transfected with either Flag-YAP1-1 or Flag-YAP1-2 into HEK293T cells as indicated. The interaction of YAP1 and PTPN14 was determined by co-immunoprecipitation and immunoblotting with anti-FLAG and anti-PTPN14. Flag-YFP was used as control. (E) L3.6-YAP1-1δ and L3.6-YAP1-2δ cells were cultured at low cell density for 3 days to accumulate YAP1 protein and then planted to 10cm dishes at high cell density and cultured for 24h. The association of endogenous YAP1 and PTPN14 was determined by co-immunoprecipitation followed by immunoblotting with anti-YAP1 and anti-PTPN14.

Supplement Figure 4

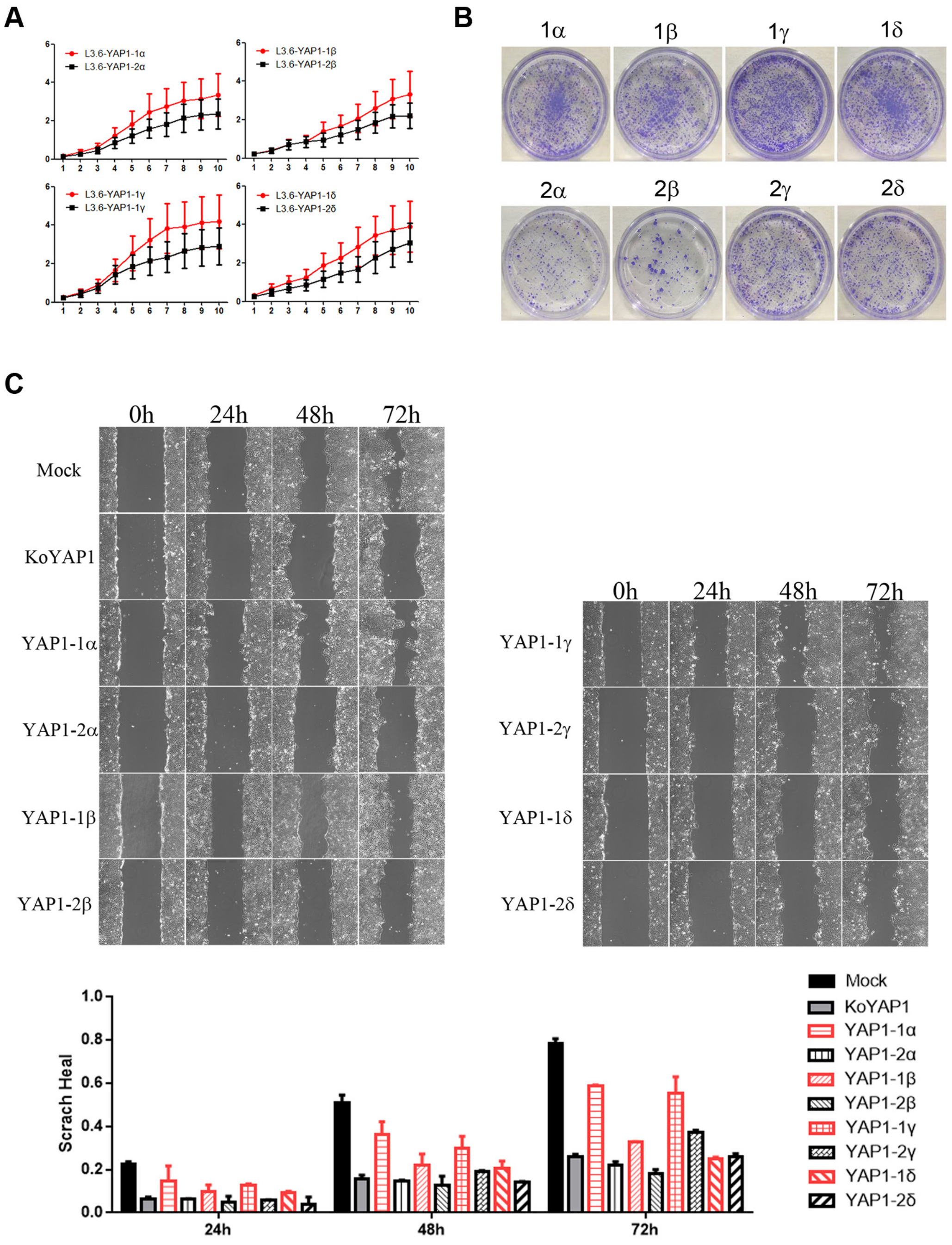

Colony formation and wounding assays of all YAP1 isoforms: (A) MTT assay was performed to determine the proliferation of L3.6-YAP1-x cells. (B) Colony formation analysis of L3.6-YAP1-x cells. (C) Wound healing assay to determine the migration of L3.6-YAP1-x cells (up) and statistical analysis (down).

Supplement Figure 5

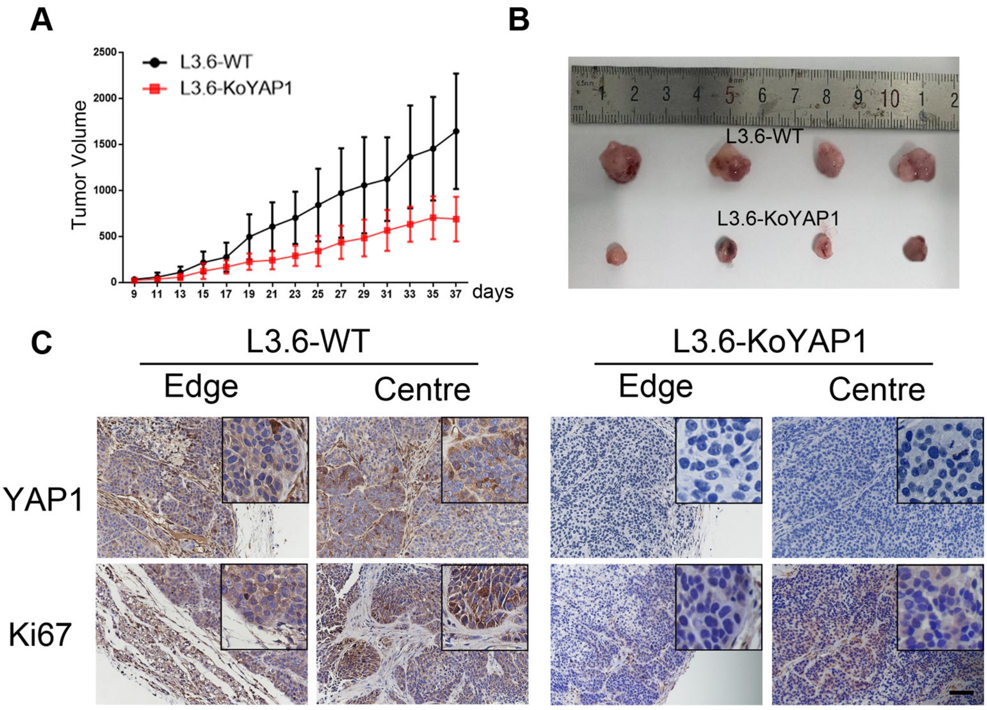

Tumor xenograft experiment: BALB/c nu/nu mice were subcutaneously transplanted with 1 x 10^6 of L3.6-WT or L3.6-KoYAP1 cell (n=4). (A) The growth curves of tumor xenografts. The tumor volume was calculated by the formula: V= (length x $width^2$)/2. (B) Representative macroscopic appearance of the tumors. (C) Tumor tissue sections were subjected to IHC analysis with YAP1 and Ki67 antibodies. Scale bar, 50μm.

Supplementary methods

YAP1 cDNA cloning and construction of other related expression, knockdown and knockout plasmids

To generate mammalian expression vectors for all 8 YAP1 isoforms, we first subcloned *YAP1-2α* (ORF=488 a.a.) from *pCMV-2xFlag-YAP1* (Addgene #19045) into the *pCI2-Flag* vector, a modified version of *pCI* (Promega, Madison, WI), at the EcoRI and SalI sites. Next, we deleted the 114-bp DNA fragment encoding the 2nd WW domain from *YAP1-2α* by overlapping PCR to obtain *pCI2-Flag-YAP1-1α*. Using the same strategy, we constructed the other full-length YAP1-1 and YAP1-2 isoforms (see Figure 1C). To facilitate the stable overexpression of YAP1 in PDAC cells, we subcloned each of the 8 YAP1 isoforms into a lentiviral expression vector. For this purpose, the cDNA insert encoding each of the YAP1 isoforms was isolated from the *pCI2-Flag-YAP1* vector by digestion with EcoRI and SalI and subcloned in frame into a modified version of the *pLenti6.3 vector* (Invitrogen, USA) at the EcoRI and XhoI sites with an N-terminal Flag tag. To clone *YAP1* cDNA encoding the TA domain into the *pM vector* (Clontech, Palo Alto, CA), the cDNAs encoding the C-termini of four *YAP1* TA domain isoforms were amplified by PCR (forward: 5'-ACCGAATTC AGTGCTCCAGTGAAACAGCC-3' and reverse: 5'-TTGGTCGACTAGCTTTCTTTATC TAGCTTGGTGGC-3'). EcoRI and SalI fragments were inserted in frame into the same sites in the *pM* vector with an N-terminal GAL4 DNA binding domain (DBD). Human cDNA encoding *TEAD1* (a.a. 101-426) was cloned into the *Tag2B* vector with an N-terminal Flag epitope tag. For knockdown of endogenously expressed LATS1 and LATS2, we used the *pLKO.1* lentivirial expression system (Sigma, St. Louis, MO, USA). The shRNA targeting sequences (listed in the 5′ to 3′ direction) were CACGGCAAGATAGCATGGATT (*shLATS1a*) and CTACTCGCCATACGCCTTTAA (*shLATS2a*). To knock out endogenous YAP1,

a CRISPR/Cas9-based vector was constructed in *pSpCas9 (BB)-2A-Puro (PX459) V2.0* (Addgene Plasmid #62988) with the guide sequence in the YAP1 5' coding region (5'-GCATCAGATCGTGCACGTCCG-3') and recommended procedures. The single-stranded oligos were annealed, phosphorylated and cloned into the BbsI sites of the PX459 backbone vector following the recommended strategy. The construct was sequenced to confirm the presence of the desired YAP1-sgRNA sequence.

Analysis on the relative expression of YAP1 isoforms

To determine the relative mRNA expression of each YAP1 isoform, the following primers were designed to amplify the cDNA fragment flanking the 2nd WW and the region of the TA domain subjected to alternative splicing: forward (5'-ATGCTGTCCCAGATGAACGTCAC-3') and reverse (5'- ATCCTGCTCCAGTGTT GGTAACT-3'). The predicted lengths of the PCR amplicons were 297 bp for YAP1-1α, 309 bp for YAP1-1β, 345 bp for YAP1-1γ, 357 bp for YAP1-1δ; 411 for YAP1-2α, 423 bp for YAP1-2β, 459 bp for YAP1-2γ and 471 bp for YAP1-2δ. Purified RT-PCR products were cloned into the pGEM-T vector. One hundred bacterial colonies were picked and seeded for minipreps followed by direct DNA sequencing. The sequencing results were compiled and analyzed to calculate the proportion of each isoform relative to the total.

To determine the mRNA expression of YAP1 and its target genes, the following gene-specific primers were used in this study:

YAP1 forward: 5'-CAAATCCCACTCCCGACAG-3'

YAP1 reverse: 5'-GTCAGTGTCCCAGGAGAAAC-3'

CTGF forward: 5'-CTTCTGTGACTTCGGCTCC-3'

CTGF reverse: 5'-ACGTGCACTGGTACTTGC-3'

CYR61 forward: 5'-CAAGGAGCTGGGATTCGATG-3'

CYR61 reverse: 5'-AAAGGGTTGTATAGGATGCGAG-3'

GAPDH forward: 5'-ACATCGCTCAGACACCATG-3

GAPDH reverse: 5'-TGTAGTTGAGGTCAATGAAGGG-3'

All values were normalized to GAPDH.
